# On the Formation of Lipid Droplets in Human Adipocytes: The Organization of the Perilipin–Vimentin Cortex

**DOI:** 10.1371/journal.pone.0090386

**Published:** 2014-02-28

**Authors:** Hans Heid, Steffen Rickelt, Ralf Zimbelmann, Stefanie Winter, Heiderose Schumacher, Yvette Dörflinger, Caecilia Kuhn, Werner W. Franke

**Affiliations:** 1 Helmholtz Group for Cell Biology, German Cancer Research Center (DKFZ), Heidelberg, Germany; 2 Progen Biotechnik, Heidelberg, Germany; German Cancer Research Center, Germany

## Abstract

We report on the heterogeneity and diversity of lipid droplets (LDs) in early stages of adipogenesis by elucidating the cell and molecular biology of amphiphilic and cytoskeletal proteins regulating and stabilizing the generation of LDs in human adipose cells. A plethora of distinct and differently sized LDs was detected by a brief application of adipocyte differentiation medium and additional short treatment with oleic acid. Using these cells and highly specific antibodies for LD-binding proteins of the perilipin (PLIN) family, we could distinguish between endogenously derived LDs (endogenous LDs) positive for perilipin from exogenously induced LDs (exogenous LDs) positive for adipophilin, TIP47 and S3-12. Having optimized these stimulation conditions, we used early adipogenic differentiation stages to investigate small-sized LDs and concentrated on LD-protein associations with the intermediate-sized filament (IF) vimentin. This IF protein was described earlier to surround lipid globules, showing spherical, cage-like structures. Consequently - by biochemical methods, by immunofluorescence microscopy and by electron- and immunoelectron microscopy - various stages of emerging lipid globules were revealed with perilipin as linking protein between LDs and vimentin. For this LD-PLIN-Vimentin connection, a model is now proposed, suggesting an interaction of proteins via opposed charged amino acid domains respectively. In addition, multiple sheaths of smooth endoplasmic reticulum cisternae surrounding concentrically nascent LDs are shown. Based on our comprehensive localization studies we present and discuss a novel pathway for the LD formation.

## Introduction

Most of the bodýs storage pool of lipids is located as lipid droplets (LDs) in adipose tissue; although almost all cell types possess LDs. The main function of LDs in cells is the regulation of lipid homeostasis and energy supply, as well as involvement in the synthesis of membranes and lipid-containing components. Increasing attention is being focused on adipogenesis, the differentiation process of fat cell formation and LD biogenesis. These processes play pivotal roles in many human diseases like fatty liver diseases, atherosclerosis, diabetes, “fatty replacements” in heart tissue, lipodystrophies, and obesity, and they have major impacts in host-pathogen pathways [1]–[11]. Despite this recognition and growing importance, the numerous studies on LDs did not yet lead to the understanding of the basic mechanisms which constitute LD generation processes. The basic processes still are little understood and enigmatic.

To gain a better insight into LD biogenesis, we used a panel of recently generated highly specific mono- and polyclonal antibodies for individual amphiphilic LD-binding proteins of the perilipin (PLIN) protein family (for new nomenclature PLIN 1-5 of mammalian perilipin family members see [12]) and discovered specific interactions of those proteins with the intermediate-sized filaments (IFs) keratins K8 and K18 in epithelium-derived cultured cells [13]. The aim of our current study was now to revisit adipocytes, i.e. non-epithelium-derived cells, and to elucidate potentially comparable molecular interactions of PLIN proteins within these cells in which vimentin is the only IF protein present.

Earlier reports, mainly working with mouse adipocytes, showed by electron microscopy (EM) that filamentous structures are abundant and seen in close vicinity of LDs [14]–[17] (for further literature see [15]). Vimentin was unambiguously localized and has been associated with LDs since the work of Franke and co-workers using mouse 3T3-L1 cells and high resolution electron and immunoelectron microscopy [15]. However the association of LDs with other filaments, microtubules and microfilaments, was excluded by these authors.

In adipocytes generally nascent LDs are thought to arise from the bilayer membrane of ER (see e.g. [2], [18], [19]). Common studies on adipogenesis conventionally use long-term treatment with adipocyte differentiation medium, mainly resulting in fully differentiated, coalesced LDs (see e.g. [15], [20]. Such differentiated cells contain huge LDs of several µm in diameter (for commonly used LD isolation procedures see [21]). We developed new, short-time adipogenic stimulation protocols for the investigation of early steps in LD biogenesis and LD accumulation which resulted in small, newly synthesized droplets with sizes in the lower and middle nm-range in diameter – and included LD surface proteins directly involved in LD biogenesis [13]. In addition brief treatment with oleic acid (OA), applied to the culture medium and in parallel to adipogenic stimulation, was tested. Hence, using our new protocols, we were able to follow endogenously formed and exogenously induced LDs simultaneously. Moreover, this new approach allowed us to obtain myriads of distinct and differently-sized (mainly small-sized) LDs in individual adipocytes. Further characterization of these small LDs, using biochemical and immunofluorescence microscopic (IFM) methods as well as electron- and immunoelectron microscopy (EM, IEM), led to the recognition of sequential LD formation steps with the involvement of multiple sheaths of smooth endoplasmic reticulum (ER) cisternae surrounding nascent LDs, which also revealed evidence for the direct interaction of PLIN proteins with vimentin. Based on these data, novel models for a LD-PLIN-Vimentin complex and for an adipose differentiation pathway are presented and discussed.

## Material and Methods

### Antibodies and reagents

The generation of highly-specific monoclonal (mab) and polyclonal (pab) antibodies against human and murine members of the perilipin (PLIN) family of LD-binding proteins and secondary antibodies were performed as recently described ([13]). Details of the antibodies used in this study are given in **Table S1**. Oleic acid (OA) complexed with bovine serum albumin (BSA; Sigma-Aldrich, Taufkirchen, Germany) was applied to the cell culture media (usually 100 µM/ml OA for 1-3 h) prior to fixing or harvesting of the cells.

### Cell cultures

Human preadipose cells (Poietics™ from visceral fat), growth media and adipocyte differentiation medium (in text and in figures also described as adipocyte induction medium, AIM) – containing insulin, dexamethasone, indomethacin and isobutyl-methylxanthine - were obtained from Lonza (Basel, Switzerland; Cat.Nos. PT-5005, PT-8002 and PT-8202).

### Electron and immunoelectron microscopy

For electron microscopy (EM), cells were grown on cover slips and fixed as previously described using glutaraldehyde and OsO_4_ - either simultaneously [22] or sequentially [13]. For detailed immunoelectron microscopical techniques see [13], [15].

### Crosslinking and immunoprecipitation

Human preadipose cells were cultivated and briefly stimulated (1–3 days) using AIM. Cell lysates were obtained by previously described nitrogen cavitation ([13]). Aliquots of the lysed material/cells were centrifuged at 1000 *g* to remove cell debris. Then the supernatant was crosslinked using BM(PEG)2 (Thermo Scientific, Schwerte, Germany) - for irreversible crosslinkage of reduced cysteins – following the suppliers recommendation protocol. A 20 mM stock solution of crosslinker was added to the cell lysate at a final concentration of 0.2 mM. Incubation was performed for 30 minutes at room temperature. For protein precipitation 4 volumes of methanol were added to the crosslinked and the non-crosslinked lysates and stored over night at –20°C. The methanol mixtures were centrifuged (16,100 *g* for 15 min at 4°C) and the resulting pellets dissolved in SDS-PAGE lysis buffer or in IP buffer (RIPA buffer). For details on RIPA buffer and IP procedure using protein G- or protein A- coated magnetic beads, for nitrogen cavitation, density gradient separation, SDS-PAGE, immunoblotting, silver staining and mass spectrometry see Heid et al. [13].

## Results

### Conventional adipogenic conversion

During characterization of antibodies, we revisited the process of adipogenesis and worked with conventional stimulation protocols and preadipocyte cells. These treatments and the differentiation of cells with adipocyte induction medium (AIM) usually took up to several weeks (cp. **Fig. S1**). By immunofluorescence microscopy (IFM) we noticed some small LDs positive for adipophilin situated between large-sized perilipin-positive LDs (not shown). This indicated that adipophilin did not seem to be completely replaced by perilipin, which was, so far, the prevailing opinion in literature on differentiating adipocytes. In electron microscopy (EM) of conventionally stimulated adipose cells, we occasionally found LDs with a bilayer membrane at the surface as well as many other LDs only revealing a monolayer membrane surface (not shown). Because in adipocytes LDs are reported to be surrounded by a monolayer biomembrane and not to possess a normal bilayer membrane, as seen in other cell organelles, we were surprised by the occurrence of both types of membranes with LDs.

### Preadipose cell stimulation and the generation of LD heterogeneities

**Figure 1:**
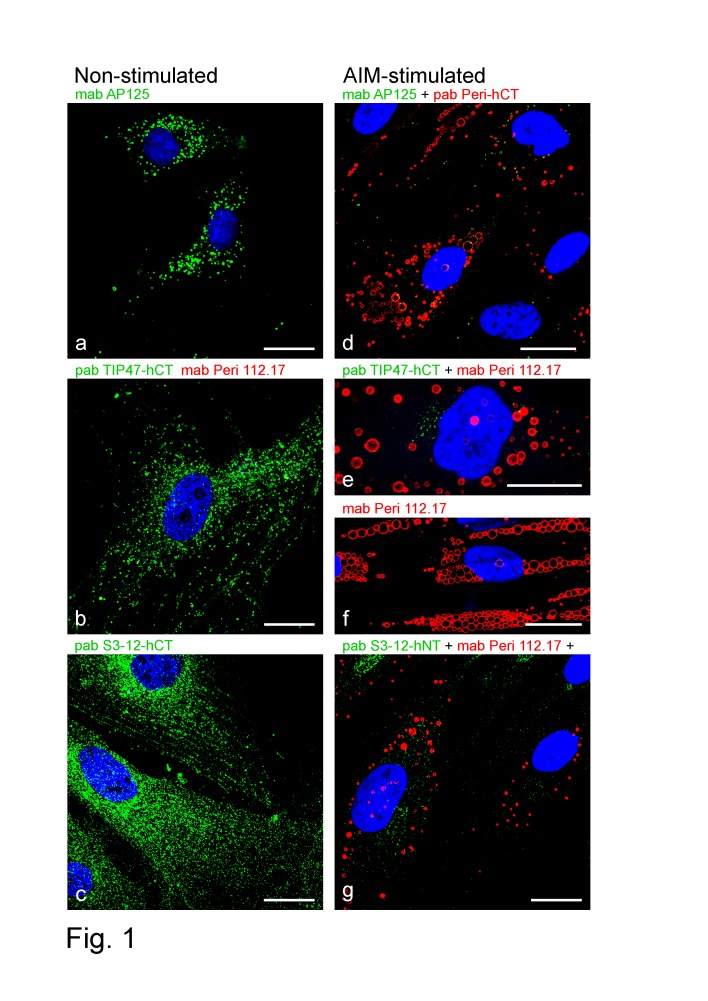
Laser scanning single- and double-label immunofluorescence microscopy showing lipid droplet (LD) labeling of perilipin proteins in untreated, non-stimulated and in briefly AIM-stimulated human preadipocytes. (a-c) Non-stimulated cells. (a) Adipophilin monoclonal antibody (mab) staining reveals many small LDs (green). (b) Positive TIP47 polyclonal antibody (pab; green) vs. negative perilipin (mab; red) staining. (c) Pab S3-12 shows many very small LDs – some can be seen like arranged and placed along rows of filaments. (d-g) Adipocyte differentiation of cells with AIM medium. 1-3 days of AIM treatment newly generates many small and medium-sized LDs, which stain with antibodies for perilipin (red). In contrast after AIM treatment, LDs positive for other perilipin (PLIN) family members are reduced in numbers and size (green). Cells still appear fibroblast-like elongated, not roundish. (d) Perilipin vs. adipophilin staining. (e) Perilipin and TIP47 double staining. (f) Perilipin localization at surface of LDs (g) Perilipin comparison with S3-12. Note, whereas antibodies specific for adipophilin, S3-12 and TIP47 show plenty of small LDs in non-stimulated cells and staining for perilipin is negative, the situation changes completely with the start of AIM stimulation. (For additional examples of small LD staining conspicuously arranged along rows of filaments, see e.g. TIP47 staining patterns obtained with PLC epithelial cells given in Fig. S5 by Heid et al., 2013 [13]). Nuclear staining was with DAPI (blue). Bars: 20 µm.

The results obtained with conventionally stimulated preadipocytes indicated that LDs within individual cells might not be as uniform as thought, but could consist of different types of droplets (see also [13], [23]). Therefore we shortened the stimulation procedures and concentrated on the early onset of LD biogenesis, i.e. situations in which very small LDs emerge and the cytoplasm of the cells was not completely occupied by large LDs. We used standardized, commercially available human preadipocytes which allowed 2–5 working passages after starting adipogenic conversion. Most cells were accessible for stimulation and contained, thereafter, numerous LDs (approximately 70–80% of all cells could be stimulated). Within freshly seeded, non-stimulated preadipose cells, detection of the PLIN proteins adipophilin, TIP47 and S3-12 was positive, but as expected, essentially no staining for perilipin was found (**Fig. 1a-c**). The elongated preadipose cells often showed – after fresh seeding and having only few cell-cell contacts - astonishingly long dendrites with plenty of positively stained tiny LDs in the cytoplasm. Adipophilin antibodies revealed many small LDs - many of them could be localized in long dendrites (example given in **Fig. S2**). S3-12 and TIP47 antibodies revealed many very small LDs – some visibly arranged along rows, possibly filaments (**Figs. 1b,c**). Perilipin antibodies applied to non-stimulated cells were negative (see e.g. **Fig. 1b**).

After 12–24 hours of AIM stimulation, i.e. shortly after starting the adipogenic conversion program, perilipin was traceable in IFM with small to medium-sized LDs (**Fig. 1d-g**). Other PLIN proteins were still positive and visible, but compared to their expression in non-stimulated preadipose cells, reduced in numbers and sizes (cp. **Figs. 1d,e,g** with **Figs. 1a,b,c** respectively).

**Figure 2:**
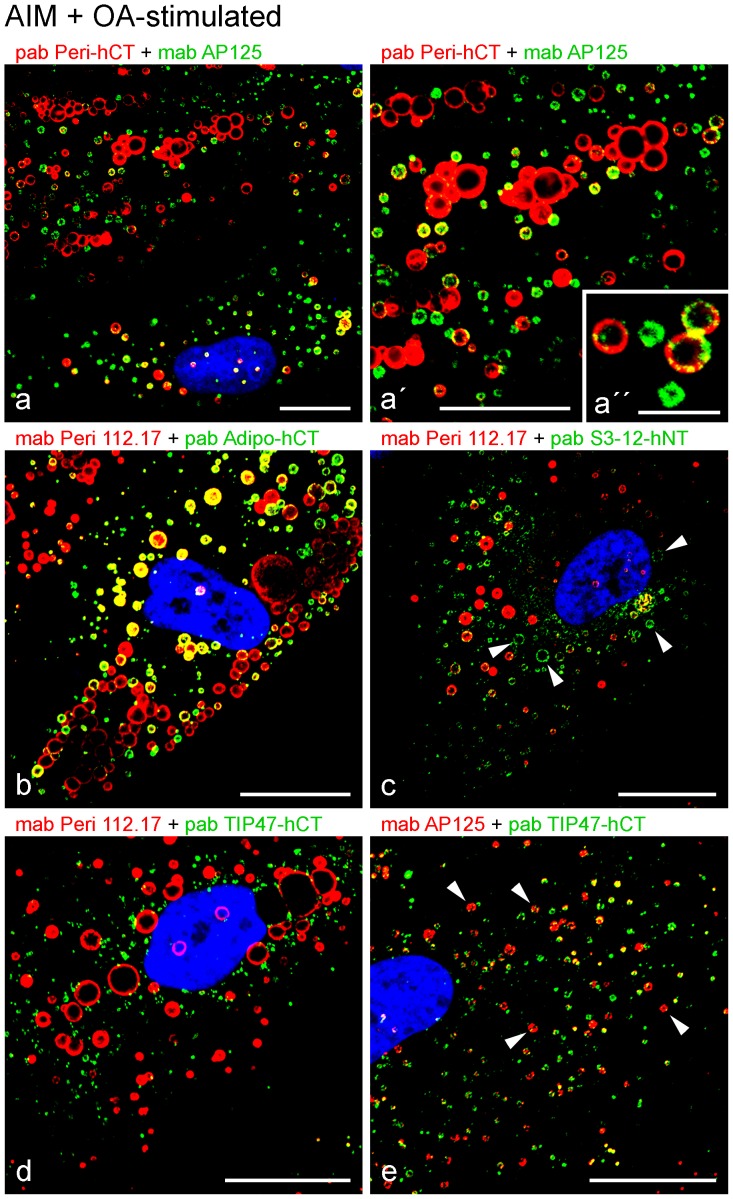
Laser scanning microscopy showing LD labeling of perilipin proteins in briefly AIM-stimulated human preadipocytes which were additionally treated shortly with oleic acid (OA). (a, a, a) AIM treatment for 1–3 days reveals many newly generated small and medium-sized LDs positively staining with perilipin antibodies (red). Additional OA-treatment for 2 hours leads to the appearance of LDs positive for adipophilin (green) in higher numbers and bigger sizes when compared to non-stimulated or AIM-stimulated cells solely (cp. Fig. 1). (a) Enlarged part taken from double staining seen in (a). (a) Enlarged part from (a) showing single color and mosaic double-stained droplets, obviously in the midst of a fusion process with involvement of different types of LD-binding PLIN proteins and with yellow, partly mixed colored LDs for co-localization. (b) A different perilipin/adipophilin antibody combination reveals a similar heterogeneous staining of LDs as seen in a-a. (c) Perilipin (red) vs. S3-12 (green) double staining. Several smaller S3-12-positive droplets are seen attaching and combining with larger perilipin-positive droplets. Many newly generated, very small and middle-sized rings of S3-12-positive droplet staining with punctate patterns can be seen by single green color staining only (examples marked by arrowheads). (d) Perilipin (red) vs. TIP47 (green) double staining. Some of the many newly appearing TIP47-positive small droplets adhere occasionally to the much bigger perilipin positive droplets. (e) Adipophilin (red) vs. TIP47 (green) double staining. Most of the droplets are visible with sizes estimated smaller than 1 µm in diameter. Some heterogeneously colored droplets are marked by arrowheads. Note, OA-treatment leads to the re-appearance of a tremendous number of small droplets positive for adipophilin, TIP47 and S3-12. Note in addition, the complexity of the PLIN staining of LDs. Different LD-binding proteins are expressed and different types of LDs appear in single cells by specific “endogenous” and “exogenous” stimulations. DAPI (blue). Bar in a: 5 µm; all other Bars: 20 µm.

Additional OA-treatment for 1–3 hours in parallel to the proceeding AIM stimulation induced the re-appearance of many LDs positive for adipophilin, TIP47 and S3-12 with overall bigger spherical droplet sizes when compared to non-stimulated or merely AIM-stimulated cells (**Fig. 2**). A variety of heterogeneous LD staining was found when antibodies specific for adipophilin were compared with antibodies specific for perilipin. Enlargements showed single-colored and complex, mosaic-stained droplets (**Fig. 2a-a, b**). With OA treatment small S3-12-positive droplets were seen attached with larger perilipin-positive droplets. Additionally, many newly generated single color-stained, small and middle-sized rings of S3-12-positive surface droplet staining with punctate patterns could be found. Such LD-surrounding rings of S3-12 did not co-localize with the perilipin labeling and had not yet been detected with such sizes in cells not AIM-stimulated and/or not OA-treated (examples marked by arrows in **Fig. 2c**). Upon additional OA-treatment only some of the many freshly appearing small TIP47-positive droplets adhered only occasionally and docked to the much bigger perilipin positive droplets (**Fig. 2d**). OA-treatment was responsible for the re-appearance of a tremendous number of small droplets positive for adipophilin and TIP47 – some single color-stained, many others in the process of combining as seen by heterogeneous labeling (examples marked by arrowheads in **Fig. 2e**). At this early point of AIM stimulation, most of the new droplets appeared smaller than 1 µm in diameter. The heterogeneously colored droplets and the different droplet sizes revealed a huge variety and complexity. We noticed, that we could “produce” different types of LDs in individual adipocytes by specific **“**endogenous” generation in combination with endocytosis and uptake of “exogenous” hydrophobic substances.

### The incidence of different types of LDs in adipose cells: “Endogenous-LDs” and “Exogenous-LDs”

**Figure 3:**
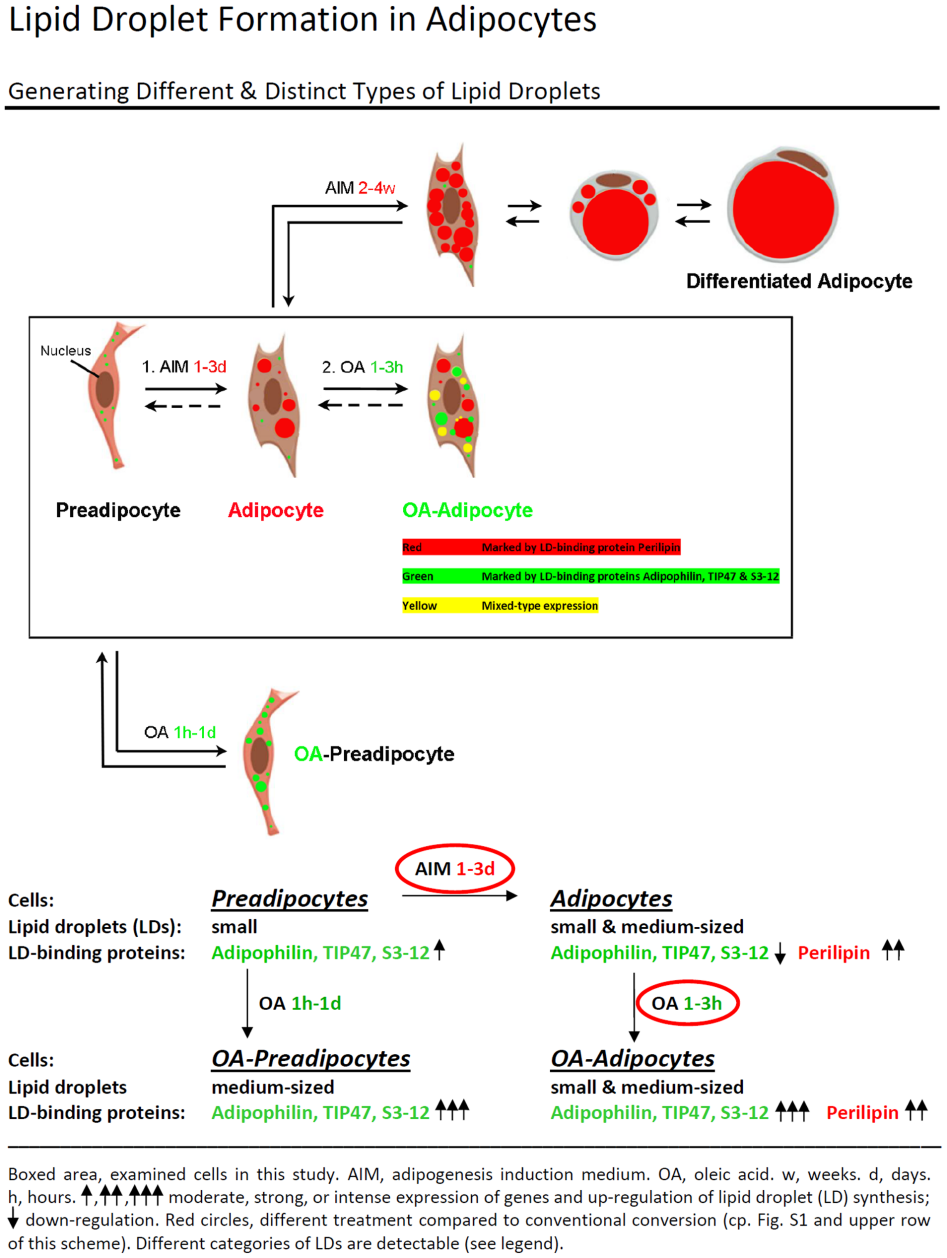
Summary scheme and brief description of stimulation methods used for adipose conversion and for the generation of different, distinct types of lipid droplets. Major treatments and involved PLIN proteins are shown. Preadipocytes containing many small LDs are not differentiated for several weeks with AIM containing media as conventionally described (top row; cp. Fig. S1), but only very briefly (1–3 days), giving rise to “Adipocytes” (boxed area). Additional, short OA-treatment leads to “OA-Adipocytes” (boxed area, right side). Treatment with OA only - without AIM stimulation - leads to “OA-Preadipocytes” (bottom). Note the huge heterogeneity of sizes and colors of LDs seen within “OA-Adipocytes”. LDs are endogenously generated at the endoplasmic reticulum and stained positively for perilipin (“Endogenous-LDs”, red). Other LDs are obtained from the exogenous uptake of OA and stained positively for adipophilin, TIP47 and S3-12 (“Exogenous-LDs”, green). Merged LDs by fusion and mixed-type expression are seen by yellow color. The backway arrows indicate possible routes of LDs during lipolysis.

The various stimulations and treatments used with the preadipose cells and the PLIN proteins involved are shown in the schematic presentation given in **Fig. 3**. Preadipocytes containing many small LDs - not stainable for perilipin, but for other PLIN proteins - were stimulated with AIM containing medium for 1–3 days only (boxed field), rather than for weeks as conventionally described which would lead to “Differentiated Adipocytes” (top row, see also Fig. S1). Such short treatment gave rise to “Adipocytes” (boxed field, middle). Additional short OA-treatment led to “OA-Adipocytes” (boxed field, right side). Incubation with OA - without AIM stimulation - induced “OA-Preadipocytes” (bottom). As indicated, a huge complexity of LD sizes and colors due to antibody staining could be detected within “OA-Adipocytes”. LDs endogenously generated at the endoplasmic reticulum (ER) stained positively for perilipin (“Endogenous LDs”). Other LDs were obtained by the exogenous uptake of OA and stained positively for adipophilin, TIP47 and S3-12 (“Exogenous LDs”). Having established these changes, we were able to regulate and to better plan for obtaining small specific LDs.

### Biochemical investigations on LD fractions and the PLIN protein–vimentin connection

**Figure 4:**
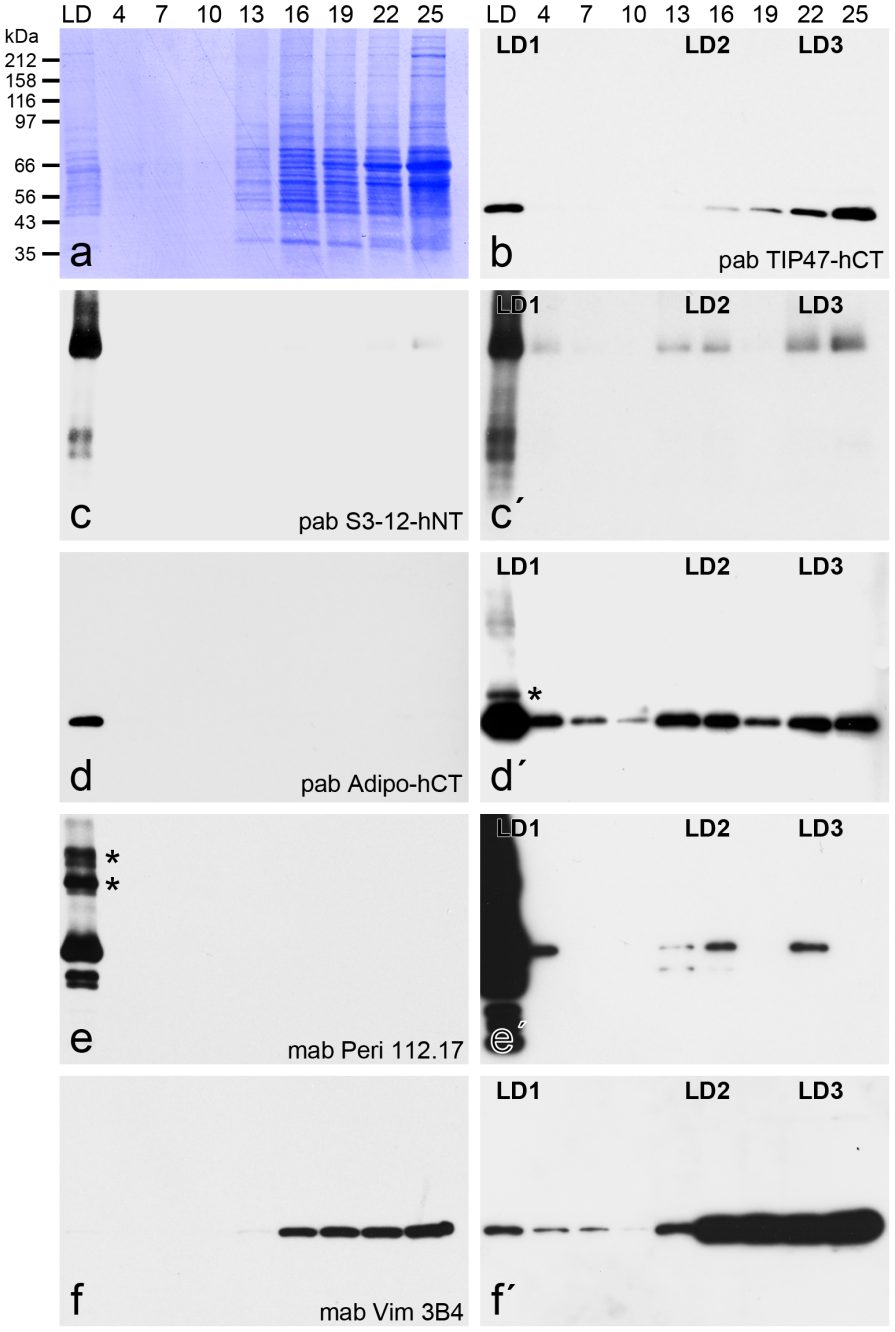
PLIN proteins and vimentin detected in different density gradient centrifugation fractions of briefly AIM-stimulated preadipocytes. (a) Fractions obtained by cell disintegration using nitrogen cavitation, iodixanol gradient centrifugation followed by SDS-PAGE separations and Coomassie blue staining are shown (cp. [13]). Separated fractions were tested with Western blotting (WB) using pabs TIP47-hCT(b), S3-12-hNT (c,c), Adipo-hCT (d,d) and mabs Peri112.17 (e,é) and Vim3B4 (f,f´). Positions of molecular weight markers are given on the left margin of (a) and fraction numbers on top of (a,b). (c,d,é,f´) represent prolonged exposures of reactions shown in (c,d,e,f) respectively. The higher molecular band reactions of adipophilin and perilipin seen in LD1 fractions are unknown modifications of PLIN proteins (asterisks in d,e). Note, the separation shown here is given with AIM-stimulated preadipocytes and not with AIM-stimulated plus OA-treated preadipocytes. Therefore only the few small (i.e. freshly endocytosed) LDs are detected. These are mostly coalesced with the bigger endogenously generated LDs and therefore preferentially found here within the LD1 fraction. Importantly PLIN proteins can be detected also in these non OA-treated preadipocytes together with vimentin within all three major LD gradient areas LD1, LD2, LD3 - including the top layer LD1.

**Figure 5:**
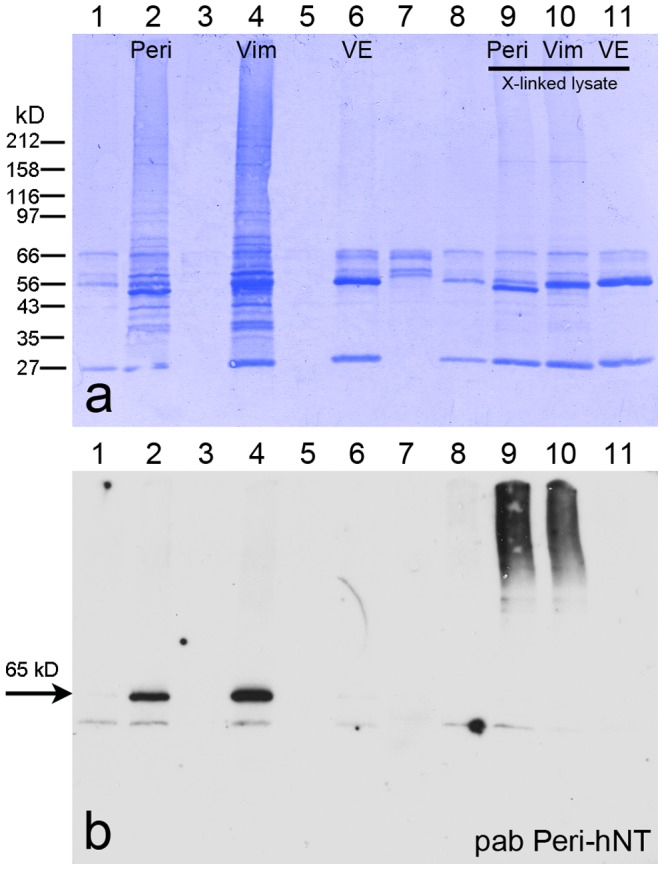
Immunoprecipitations using perilipin and vimentin antibodies and briefly AIM-stimulated preadipocytes. Mabs Peri112.17, Vim3B4 and control VE-cadherin were used. Immunoprecipitations (IPs) were analyzed by SDS-PAGE and CB staining (**a**) and corresponding WB using pab Peri-hNT (**b**). Lane 1: control unspecific binding of cell lysate to beads, i.e. magnetic beads after incubation with lysate and PBS washing; lane 2: bound lysate material of perilipin antibody beads; lane 3: control unbound supernatant of perilipin antibody beads; lane 4: bound lysate material of vimentin antibody beads; lane 5: control unbound supernatant of vimentin antibody beads; lane 6: bound lysate material of VE antibody beads; lane 7: control unbound supernatant of VE antibody beads; lane 8: control magnetic beads after incubation with crosslinked lysate; lane 9: bound crosslinked lysate material of perilipin antibody beads; lane 10: bound crosslinked lysate material of vimentin antibody beads; lane 11: bound crosslinked lysate material of VE antibody beads. Positions of molecular weight marker proteins are given on the left margin. Of importance, perilipin and notably vimentin antibodies precipitate and co-precipitate perilipin (**lanes 2,4;** perilipin position at 65 kD is marked at left margin by an arrow) in contrast to the negative control antibody (**lane 6**). In addition, similar positive reactions are obtained with crosslinked lysate by detecting crosslinked perilipin (**lanes 9,10**) whereas the control antibody shows no reaction with crosslinked material at all (**lane 11**).

With further experiments, LD-binding proteins of “Adipocytes” and “OA-Adipocytes” were biochemically separated and characterized (examples shown in **Figs. 4** **,** **5**). Samples obtained by density gradient centrifugation were tested by Western blotting. PLIN proteins could be detected within all three major LD gradient areas LD1, LD2, LD3 - including the top layer LD1 – always in conjunction with the intermediate filament protein vimentin. This specific interaction of PLIN proteins with vimentin was confirmed by immunoprecipitations (IPs) and Western blotting. Perilipin antibodies as well as vimentin antibodies precipitated and co-precipitated perilipin and crosslinked perilipin (**Fig. 5**). These results were confirmed further by mass spectrometry (MS) analysis using proteomic dissection of complete gel lanes - from top to bottom - of SDS-PAGE separated proteins of the IPs (see **Fig. S3**, **Tables S2a,b**). Vimentin was co-precipitated by a perilipin antibody and vice versa perilipin was precipitated by a vimentin antibody. Interestingly the perilipin antibody precipitated specifically the N-terminal head sequence domain of vimentin (MS results obtained within sample A6; **Table S2a**). In addition, by MS detection the same known LD-binding proteins could be co-precipitated by perilipin antibodies as well as by vimentin antibodies, namely S3-12, TIP47, 100 kD coactivator protein and Rab-18 (**Figs. S3b,c**). In other experiments, we detected also adipophilin with “OA Adipocytes” and IPs using perilipin antibodies followed by Western blotting in addition to perilipin, vimentin and TIP47 (not shown).

### Distinct perilipin localization patterns

**Figure 6:**
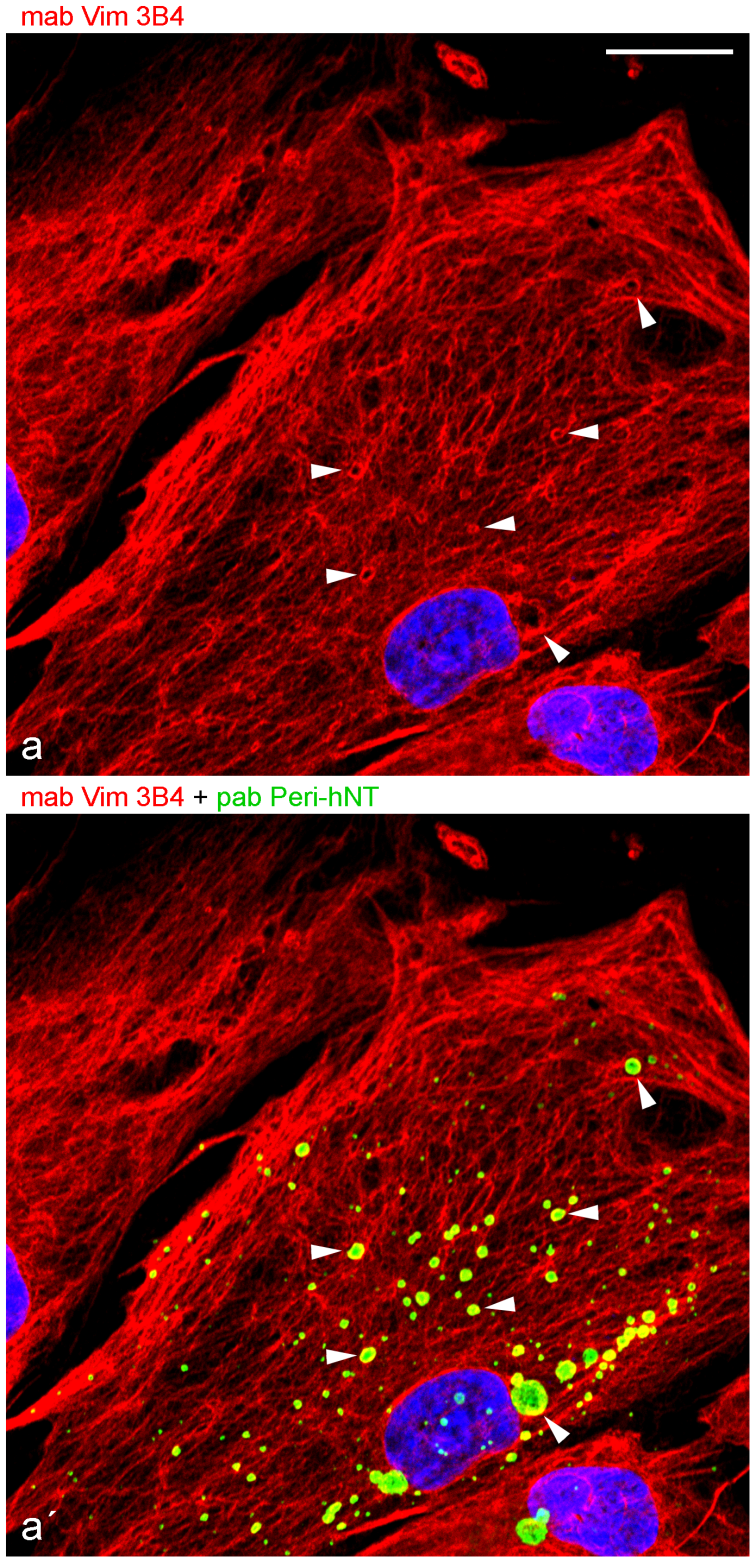
Laser scanning microscopy showing comparison of vimentin and N-terminal specific perilipin antibodies using briefly AIM-stimulated human preadipocytes. (a) Mab Vim3B4 reveals a dense filamentous network with additional prominent ring- and cage-like structures (red staining; some examples of cages are marked by arrowheads). (a) Double-staining of mab Vim3B4 (red) with pab Peri-hNT (green) unambiguously shows that these cages of intermediate-sized filaments (IFs) are marked by perilipin staining. DAPI (blue). Bar: 20 µm.

Vimentin was formerly described in association with LDs (for literature see “Introduction”) and we now found, by density gradient separations and IPs, an association of perilipin with vimentin. In briefly AIM-stimulated human preadipocytes we performed IFM and compared by double staining vimentin and PLIN using the respective antibodies (**Figs. 6** **,** **7** **,** **8**). Vimentin revealed the normal densely packed filamentous pattern within the cytoplasm. In addition, prominent ring- and cage-like structures could also be detected. The interior of these cages of surrounding vimentin could be counterstained by N-terminal specific perilipin antibody (**Fig. 6**).

**Figure 7:**
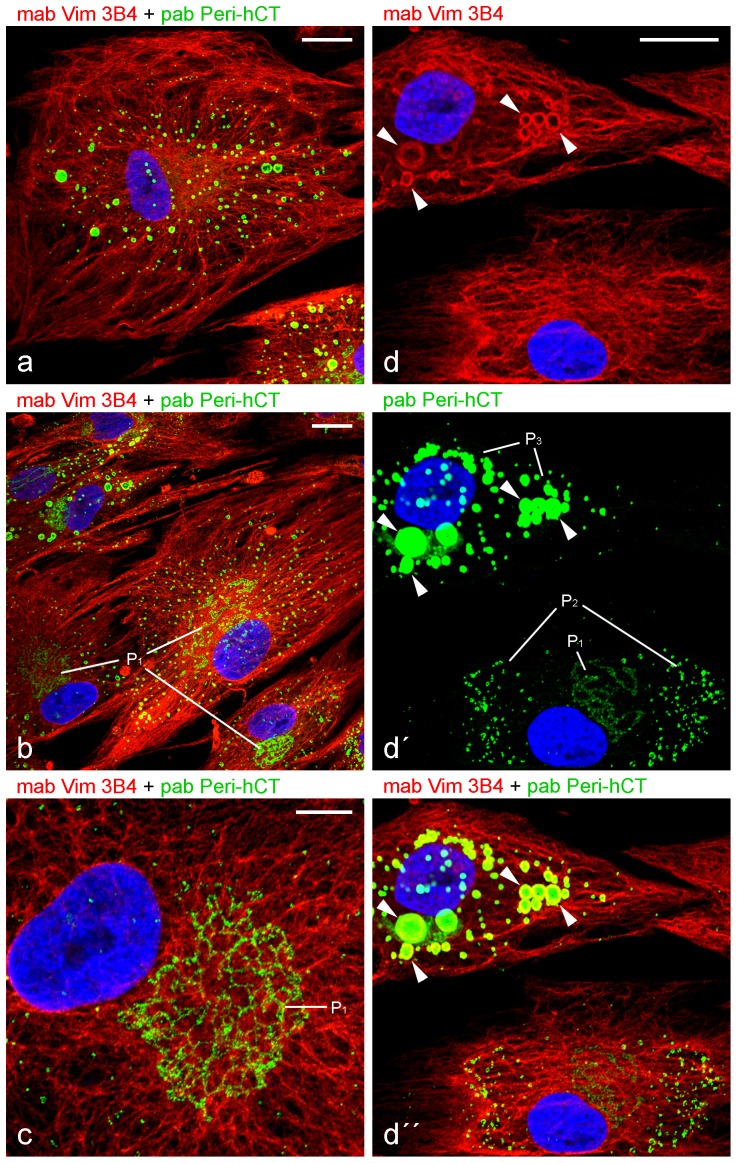
Laser scanning microscopy showing comparison of vimentin and C-terminal specific perilipin antibodies using briefly AIM-stimulated human preadipocytes and detection of different perilipin staining-patterns. (a) Pab Peri-hCT and mab Vim 3B4 reveal similar staining as seen with alike antibodies (cp. Fig. 6a). (b) At closer inspection, pab Peri-hCT often shows additional localization. Fine punctated rows of very small droplets can be seen on perinuclear distinct winded structures (perilipin staining pattern P1). (c) Enlarged perinuclear area of a cell starting adipocyte conversion. Many, tiny lipid droplets in the cytoplasm, can be seen especially at perinuclear structures (pattern P1) and in close association with the vimentin IF network. (d,d,d) Different stages of adipocyte conversion are present within the two neighboring cells shown. In the cell in the lower part of the pictures, conversion appears to begin, showing 2 different perilipin patterns - many tiny droplets binding in rows on winded perinuclear structures (pattern P1; cp. b,c) and plenty of small droplets with sizes estimated below 1 µm in diameter and distributed mainly above the perinuclear region in the cytoplasm (pattern P2). In the advanced conversion stage of the cell shown in the upper part of the picture, fairly well accomplished, bigger LDs are seen distributed all over the cytoplasm and surrounded by vimentin cages (pattern P3, examples of vimentin cages shown by arrowheads). Note, by increasing the time for AIM stimulation, we noticed an increased P3 pattern paralleled by decreasing P1 and P2 patterns. By conventionally longer AIM treatment (1–3 weeks), the P3 pattern could be seen almost exclusively in all differentiated cells. DAPI (blue). Bars in a-c: 20 µm. Bar in d: 10 µm;

**Figure 8:**
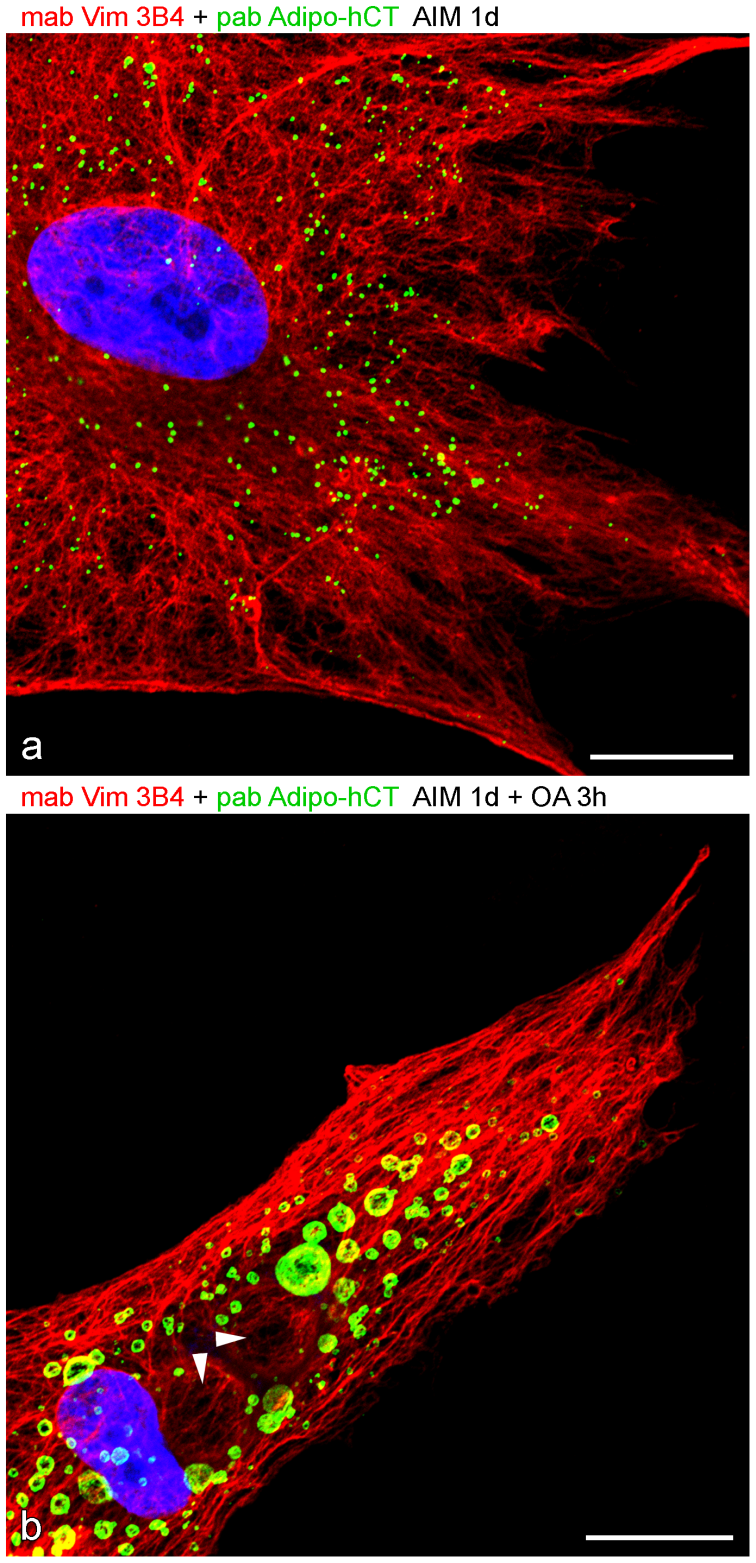
Laser scanning microscopy showing comparison of vimentin and adipophilin antibodies in AIM-stimulated compared to AIM-stimulated plus shortly OA-treated human preadipocytes. (a) With short AIM stimulation for one day many tiny LDs positively stained for adipophilin can still be seen within the dense vimentin meshwork. (a) Additional short OA treatment for 3 hours immediately leads to exogenously derived, larger adipophilin positive droplets. Arrowheads mark two huge, vimentin surrounded cage-like structures. These are probably perilipin containing droplets (cp. Fig. 7d,d) which have not yet been amalgamated by the new exogenously generated adipophilin droplets. DAPI (blue). Bars: 20 µm.

The inner sides of the cage-like vimentin structures could be tagged again when we applied a different, a C-terminal specific perilipin antibody (**Fig. 7a** **;** cp. **Fig. 6**). At closer inspection additional and different perilipin structural formations and patterns could be detected with this antibody as well. Finely punctated rows of very small droplets were seen at distinct perinuclear winded structures (Perilipin staining pattern “P1” in **Figs. 7b,c**). Tiny lipid droplets in the cytoplasm could be localized, especially at perinuclear structures and in close association with the vimentin IF network. Different stages of adipogenic conversion could be followed by the changing perilipin staining patterns “P1-P3” in **Figs. 7d-d**. Within the cell shown in the lower part of the pictures, early conversion seemed to take place with the pattern “P1” exhibiting tiny droplets associated on winded perinuclear structures. Obviously more stage-advanced than “P1”, was pattern “P2”, revealing plenty of small droplets, somewhat bigger than those from “P1”, but still with estimated sizes of less than 1 µm in diameter and with a considerable greater distribution in the cytoplasm. Within the most advanced conversion pattern “P3” of the cell shown in the upper part of the pictures series, fully grown and/or coalesced bigger LDs were seen surrounded by vimentin cages allotted throughout the whole cytoplasm of the cell.

### The re-appearance of large adipophilin-positive droplets upon OA treatment

Further comparison showed many, very small LDs positively stained for adipophilin in the midst of a dense vimentin meshwork after short AIM simulation (**Fig. 8a**). Additional OA treatment for “OA-Adipocytes” almost immediately launched numerous exogenously derived, larger adipophilin-positive droplets (**Fig. 8b**).

### Electron microscopy for studying structural details in LD biogenesis

**Figure 9:**
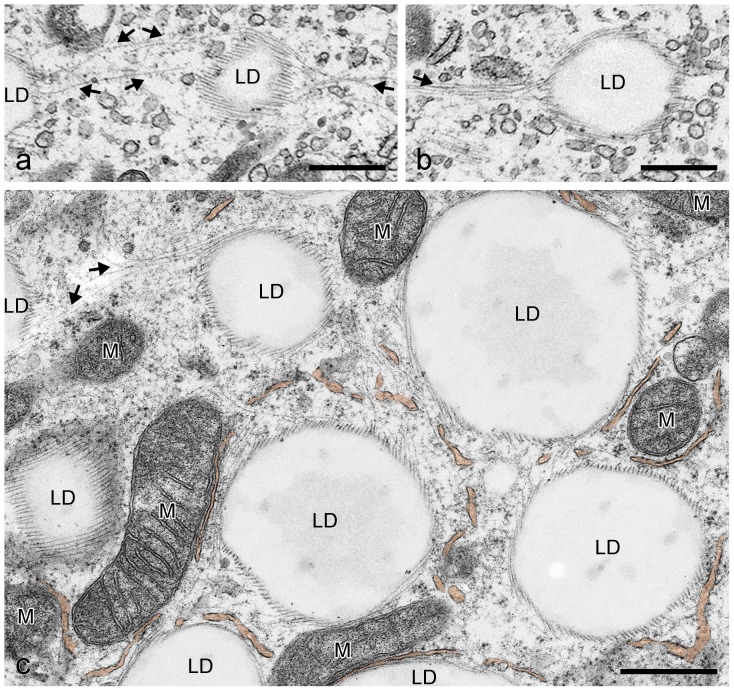
Electron micrographs showing the association of vimentin filaments with the forming of LDs after brief AIM stimulation of human preadipocytes. (a) Grazing section of two very small LDs (approx. 200 nm in diameter) surrounded by numerous vimentin IF bundles of the “wide-spacing type” with approximately 30–50 nm spacing distance (cp.[15]) and with interconnection of LDs by filaments (arrows). (b) Similar small LDs associated with filaments as seen in (a). (c) Survey electron micrograph showing ordered arrays of vimentin bundles at the surface of LDs. Some LDs can be seen interconnected by IFs (arrows). First evidences of specific assembly of smooth ER tubules forming cisternae sheaths and layers can be noticed (brown-colored system of tubules). Note, at this stage of adipocyte differentiation mitochondria (M) are not directly associated to these forming small LDs. Bars: 0.50 µm.

**Figure 10:**
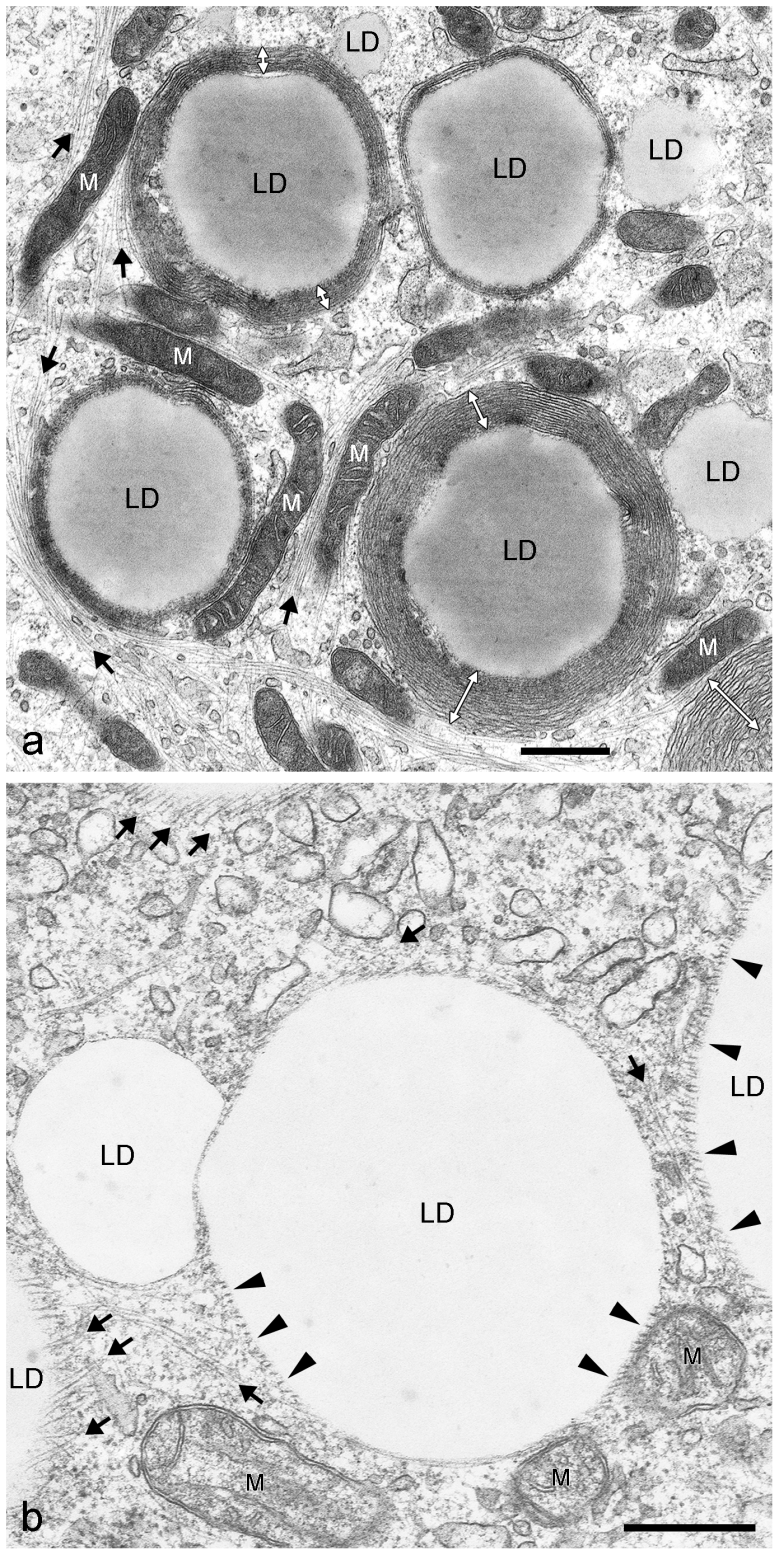
Electron micrographs comparing briefly AIM-stimulated versus briefly AIM-stimulated plus OA-treated human preadipocytes. (a) Short AIM stimulation and simultaneous fixation method, often lead to the appearance of small LDs (approximately 1 µm in diameter) which are completely surrounded by multiple cisternae and layers of smooth ER (white double arrows). Smaller LDs of 0.2– 0.5 µm in diameter without such ER layers can also be seen (right side). Embedded in a meshwork of filaments (black arrows) are mitochondria (M) in the neighborhood of these ER layers with no direct contact to LDs. (b) Addition of OA to the cell media for one day leads to the decay of the droplet surrounding ER layers (here shown with sequential fixation method). Arrowheads mark periodically arranged vimentin cage-like structures seen directly in contact with the LD surface. These regular arrays of anchored IFs have been described already by Franke et al. [15]. Several IF bundles within the cytoplasma and with connections to LDs are marked by arrows. Note, with both treatments and fixation methods, mitochondria (M) are not seen in direct contact to the LDs. Bars: 0.50 µm.

**Figure 11:**
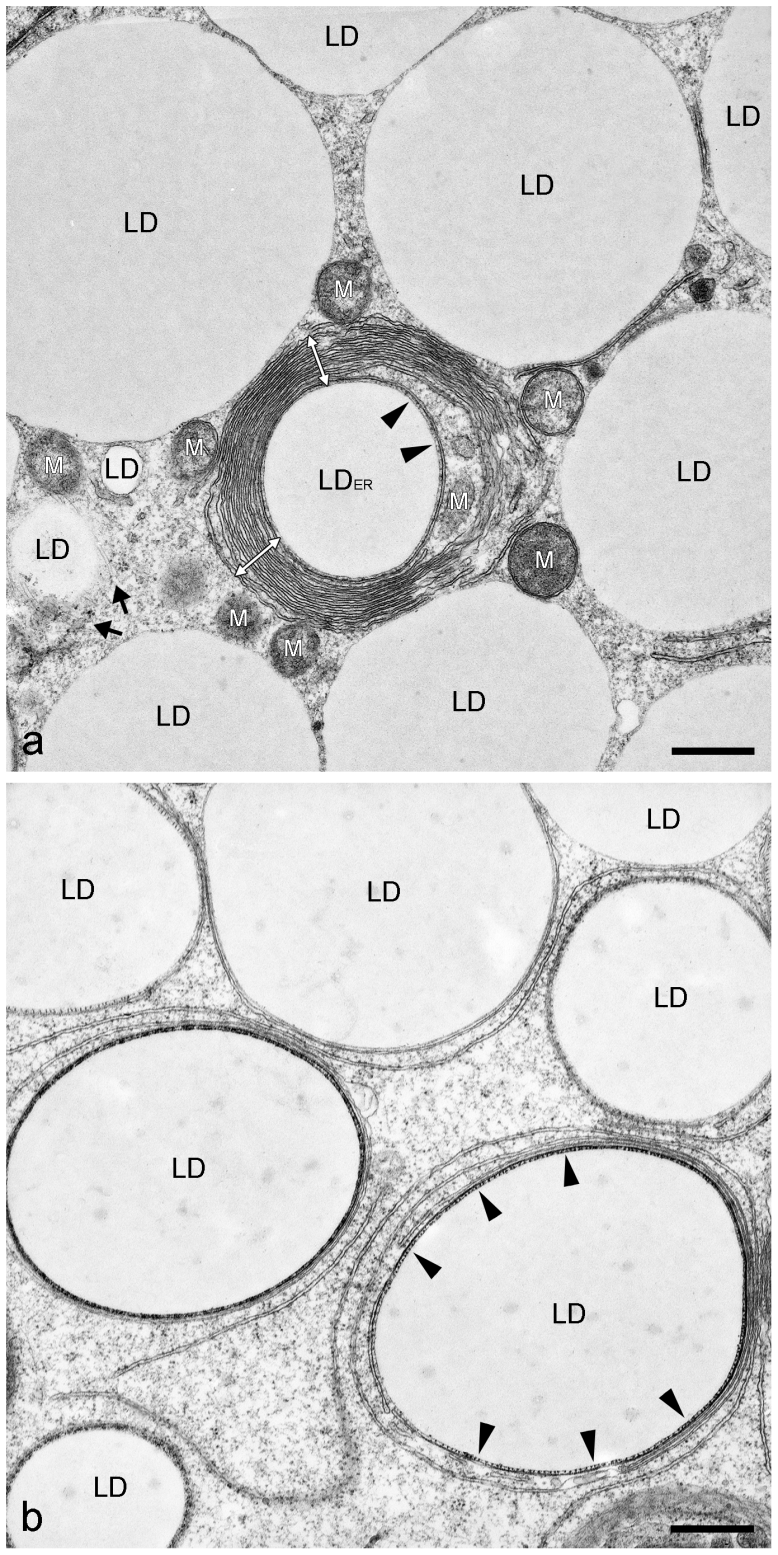
Electron micrographs showing special associations of LDs and ER within briefly AIM-stimulated human preadipocytes. (**a**) Small and characteristic LDs can be found by short-time adipose conversion showing droplets with several flat layers and cisternae of smooth endoplasmic reticulum (LD_ER_, white double arrows; simultaneous fixation method). Occasionally, very regularly spaced dots of vimentin filaments can be seen at the surface of such droplets sandwiched between droplets and ER (black arrowheads). In addition, smaller LDs of 0.2– 0.5 µm in diameter without such surrounding ER layers - but with interaction of vimentin IFs (marked by black arrows; left side) – and larger, fully accomplished LDs of several µm in diameter with mitochondria (M) in the neighborhood can be seen. (**b**) The regular pattern of vimentin arrays at the surface of LDs can be recognized best where fewer and less densely packed ER sheaths surround the droplets (black arrowheads), which might constitute a specific stage of adipogenesis. In this situation LDs seem to be matured, i.e. grown enough in size and the multiple layers of ER cisternae are just about to be released. Note, such EM pictures suggest that different stages and types of LDs in differentiating adipocytes can be pictured and characterized. Bars: 0.50 µm.

In general, the resolution of IFM pictures is not sufficient to gain detailed information on LD surfaces and LD surroundings. Therefore, we applied electron microscopy (EM) methods to extend our scope of morphological knowledge. First, we prepared electron micrographs of briefly AIM-stimulated preadipocytes and detected in grazing and tangential sections many very small LDs (approx. 100–500 nm in diameter) in contact with numerous IF bundles (**Fig. 9a-c**). The ordered arrays of the typical 7–12 nm filaments of vimentin were seen with bundles of the “wide-spacing” type with approximately 30–50 nm space distance (cp. [15]). Small LDs were also seen to be interconnected by IFs (arrows in **Figs. 9a,c**). Importantly, first evidences of sheath assembly and forming of smooth ER cisternae encircling LDs could be detected (system of ER tubules highlighted in brown color in **Fig. 9c**). A next step in differentiation and LD development is shown in **Fig. 10a**. Here, small LDs were completely surrounded by multiple concentric layers and sheaths of smooth endoplasmic reticulum (ER) cisternae. These very flat ER cisternae were found to be non-fenestrated and, with further development, the numbers of the onion ring-like arranged sheaths sometimes were seen culminating up to more than 10 in numbers. The maximum number of ER sheaths we counted was 16. The size of the LDs within complete ER cisternae was approximately 0.5–1 µm in diameter. The remaining filaments between LDs and ER sheaths were highly condensed to “high density spacing” vimentin and could not be discriminated within an overview picture (for better recognizable examples of regularly spaced vimentin filaments sandwiched between LD and ER sheaths, see later **Figs. 11** **,** **14** **)**. In **Fig. 10a**, mitochondria and a meshwork of filaments were seen in the neighborhood of those LD-ER complexes, but these had, in no case, direct contact to LDs. In cases where the AIM-stimulated cells were additionally treated with OA, the droplets surrounding sheaths of ER cisternae disappeared, decayed or became re-arranged into a meshwork of tubules of smooth ER distributed throughout the cytoplasm. Periodically arranged vimentin filaments were seen, again, loosely in contact and connecting LDs with “wide-spacing” type arrangements at the LD surfaces (**Fig. 10b**). Only in some cases were mitochondria found in the proximity – but still not directly bound - to LDs (e.g. see lower right side in **Fig. 10b**). Other situations and examples of electron micrographs showing special associations of LDs and ER are presented in **Fig. 11**. Small and modestly-sized characteristic LDs could be found by short-time adipose stimulation, with droplets showing several flat sheaths and multiple rings of smooth ER cisternae (designated LD_ER_). Regularly spaced dots sandwiched between droplets and ER were identified at the surface of such droplets (**Fig. 11a**, black arrowheads; cp. vimentin localization described [15]). In addition, smaller LDs of 0.2– 0.5 µm in diameter without such surrounding ER layers - but with interaction of filaments – could be seen. Also, many larger, seemingly fully developed LDs of several µm in diameter - notably without any discernible membrane but in contact with some adjacent mitochondria, were detectable. The regular dot pattern of vimentin seen in cross sections, the arrays at the surface of LDs could be recognized best in situations where fewer and less densely packed ER sheaths surrounded the droplets (black arrowheads in **Fig. 11b**). Overall, such EM pictures showed that EM is probably a must in LD research for the discrimination of different stages and different types of LDs during adipogenic stimulation and LD development, especially for receiving such subtle structural details.

### The localization of perilipin and adipophilin in adipose cells by immunoelectron microscopy

**Figure 12:**
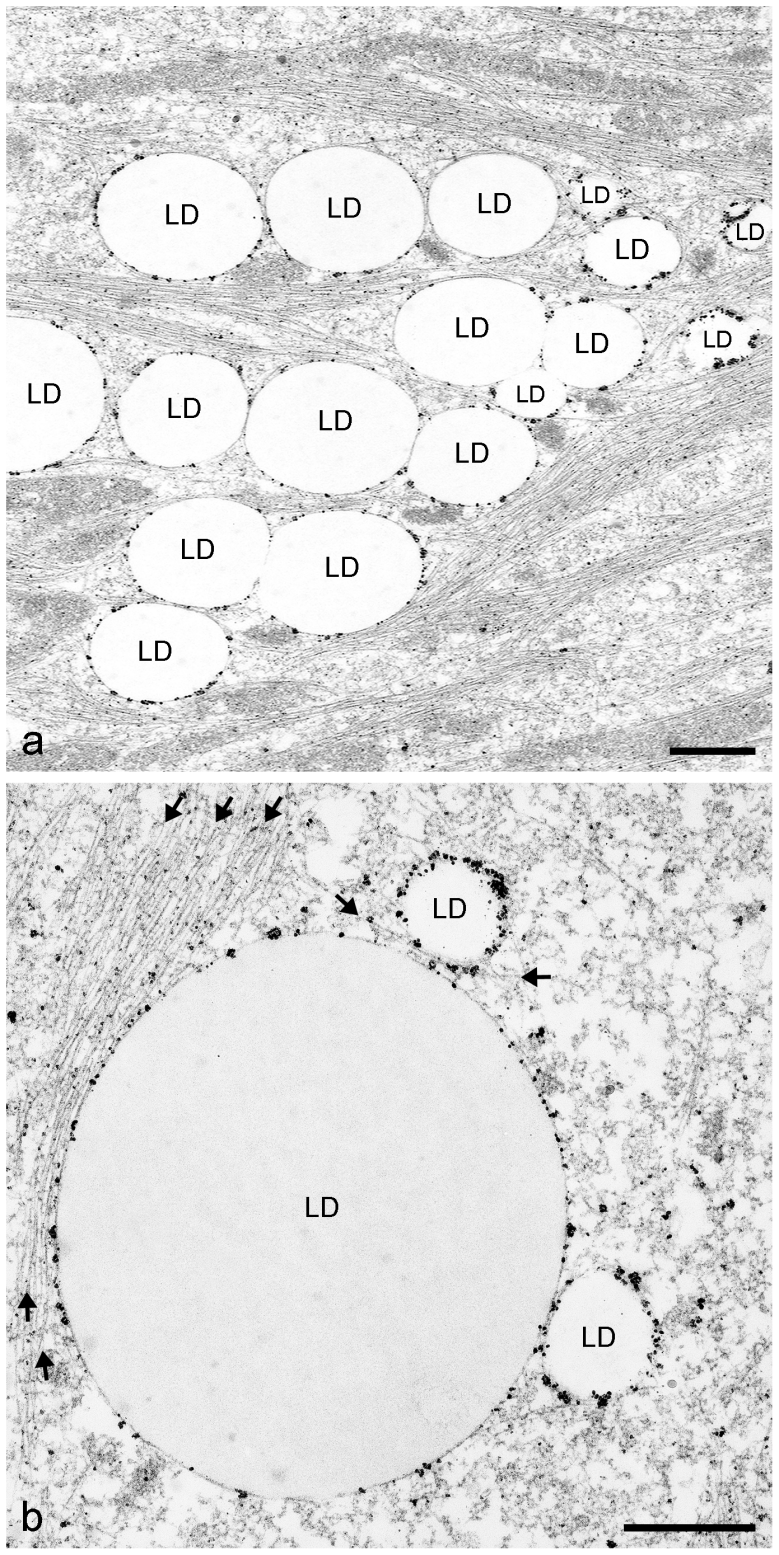
*Immunoelectron microscopic localization of perilipin in briefly AIM-stimulated human preadipocytes.* (**a**) Shown are groups of LDs positive for perilipin by nanogold-label and silver enhancement. Localization was with mab Peri112.17. The LDs are seen closely associated and anchored with IF bundles. (**b**) Enlarged perilipin labeling with two small LDs (approximately 300–400 nm in diameter) approaching a big LD (approximately 2.0–2.5 µm in diameter) for combining and coalescence. Bundles of intermediate-sized filaments are associated to grains of the immunolabeled perilipin (arrows). Bars: 0.50 µm.

**Figure 13:**
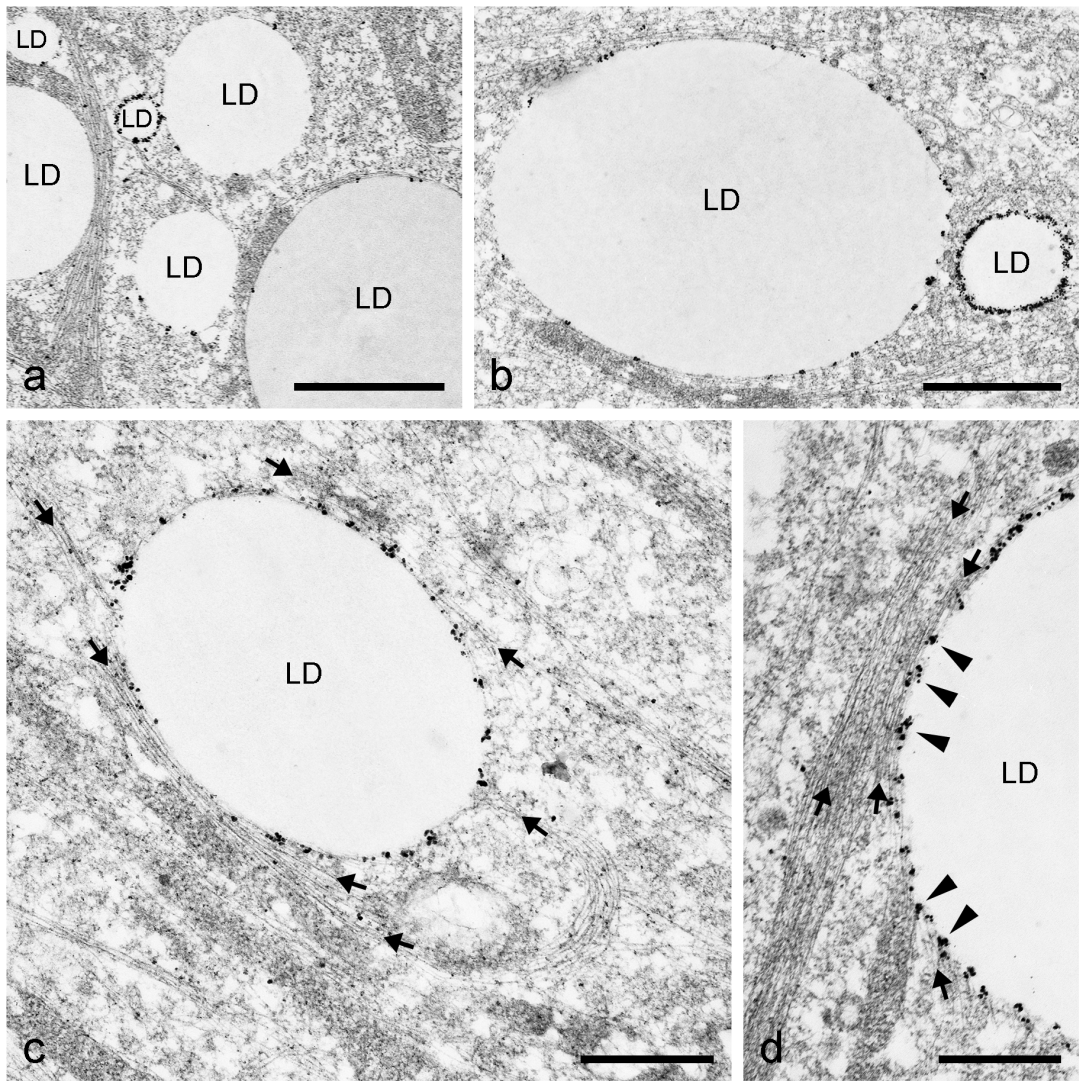
Immunoelectron microscopic localization of adipophilin in briefly AIM-stimulated and OA-treated human preadipocytes. (a) Cells reveal besides perilipin-positive also adipophilin-positive LDs. Localization was performed with mab AP125. A few positive, mostly smaller LDs are seen as well as plenty of non-labeled, larger LDs. Almost all LDs are closely associated and anchored with IF bundles. (b) A small, strongly immunolabeled LD is seen approaching a big, scarcely labeled LD. These LDs are obviously at the rim of combining. (c,d) Enlargements with details of filament attachment and immunolabeling sites which were highlighted by arrows (vimentin IFs) and arrowheads (adipophilin immunoreaction). Bars: a,b: 1 µm; c,d: 0.50 µm.

Briefly, AIM-stimulated and OA-treated preadipocytes revealed perilipin positive LDs by immunoelectron microscopy (IEM) with nano-gold-label and silver enhancement. The LDs were seen closely associated and anchored with IF bundles suggesting direct linkage with perilipin (**Figs. 12a,b**). By treatment with OA, some LDs negative for perilipin staining could be detected in the midst of many filament associated perilipin-positive LDs (see droplets designated “LD-exo” in **Fig. S4**). Using IEM and adipophilin antibodies, positively labeled LDs were seen only in few numbers, mostly smaller-sized LDs (**Fig. 13a**). LDs, either labeled or not-labeled by adipophilin, were seen closely associated and anchored with IF bundles. In **Fig. 13b** a small, strongly adipophilin-labeled LD is seen in association a scarcely labeled bigger LD. These two LDs seem to be at the begin of combining and coalescing. Enlarged pictures with details of filament attachment directly at adipophilin immunolabeling sites are highlighted in **Figs. 13c,b**. Overall the IEM results suggest a direct neighborhood and linkage of PLIN proteins with vimentin filaments.

**Figure 14:**
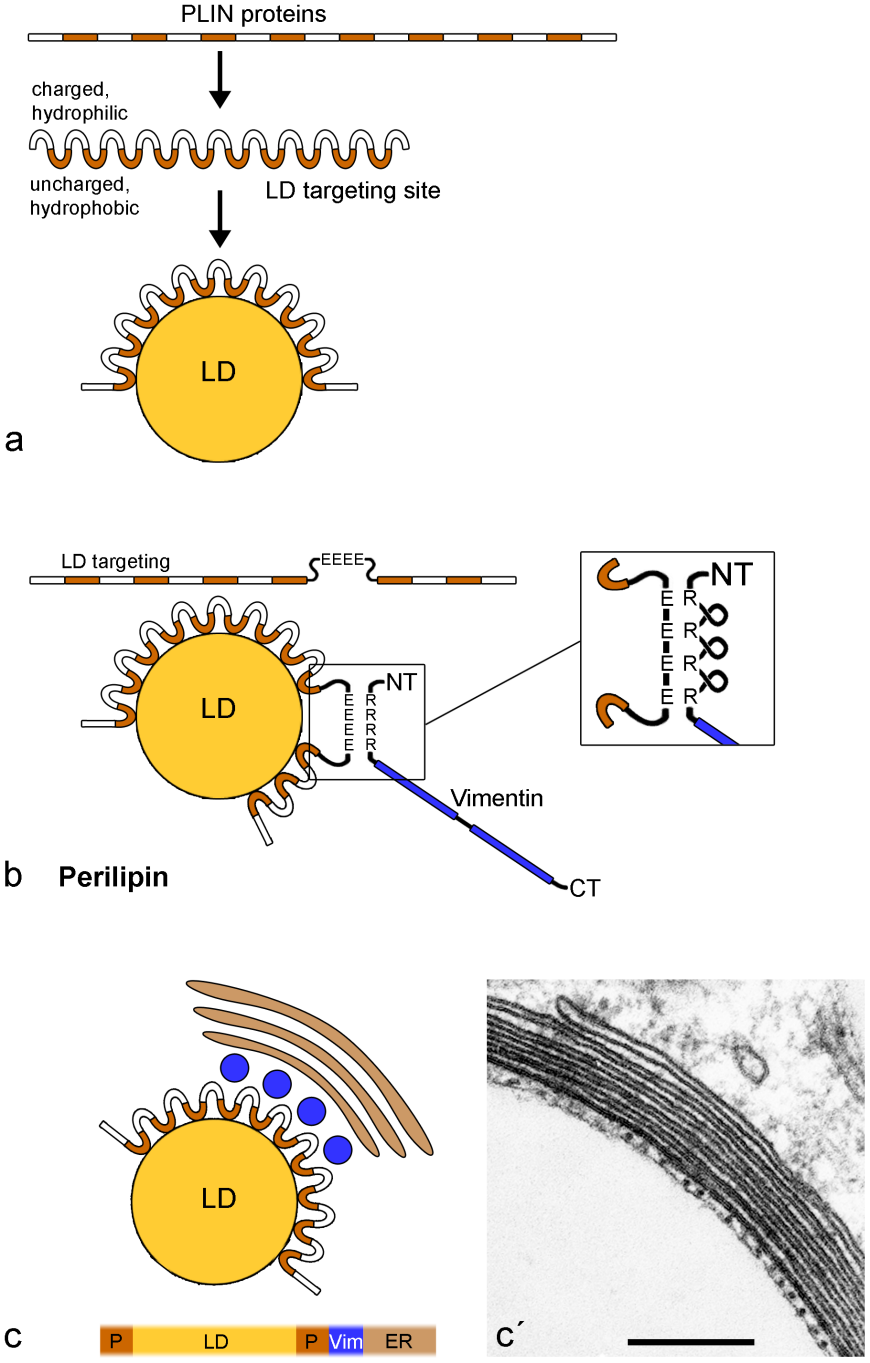
The LD-PLIN-vimentin model. (**a**) Specific hydrophobic interactions are thought to be responsible for the general binding of PLIN proteins to surfaces of LDs (cp. [13]). (**b**) Perilipin exhibits these LD targeting sites too. In contrast and uniquely compared to the other PLIN proteins, perilipin owns no helical domains at the C-terminus, but an additional acidic **E-rich** domain (see alignments given in [13], [43]). The possible interaction based on differently charged domains of perilipin and vimentin is presented. For details and for the implications of the proposed binding with the basic **R-rich** head region of vimentin see results and discussion. (**c**) A schematic summary of immunolocalization and of spatial arrangement in the proximity of emerging nascent LDs is depicted (not true-to-scale). Perilipin binds to the surface of LDs due to hydrophobic – hydrophilic short sequence domains. In a next layer, vimentin is attracted by perilipin and wraps the LDs tightly in “cage-like” spherical structures, followed by multiple concentric layers of smooth ER cisternae. This arrangement is delineated in a simplified bar-illustrated transversal section (bottom). (**c**) The schematic scenario shown in (**c**) is illustrated with a corresponding EM picture showing sheaths of non-fenestrated ER cisternae, regularly spaced dots of transversal sectioned vimentin IFs and a small rim of PLIN proteins directly bound to the LD surface. Abbreviations: E  =  glutamic acid; R  =  arginine; NT  =  N-terminal; CT  =  C-terminal; P  =  perilipin; Vim  =  vimentin; ER  =  endoplasmic reticulum. Bar in (**c**): 0.20 µm.

### Perilipin – the linker of LDs and vimentin filaments

PLIN proteins are characterized by alternating patterns of lipo- and hydrophilic sequence domains. Specific hydrophobic interactions are thought to be responsible for the general binding of PLIN proteins to surfaces of LDs (**Fig. 14a**; cp.[13]). Here, as a summary of our results, we present a model for the specific perilipin-vimentin binding (**Fig. 14b**). This proposed, charge-based binding of the two proteins - seen in EM clearly in direct vicinity - possibly functions via the binding of the acidic E-rich domain of perilipin (containing 18 acidic amino acids (aa) within a short stretch; human perilipin aa-position 292–321) and as counterpart, the basic R-rich domain of vimentin (i.e. the N-terminal “head” of vimentin containing 12 basic arginines (R) distributed in the midst of 27 annotated serine phosphorylation sites; human vimentin aa-position 2-95. Importantly, no acidic aa is found within this vimentin stretch). Because the 12 arginines of vimentin are found within the sequence domain rather equally distributed, this part of the vimentin head region might have to be compacted and twisted by loops for optimal fitting of the **E-rich domain** of perilipin (see right side of **Fig. 14b**). For arguments and advantage of such charge-based linkages see “Discussion”. The spatial arrangement within the proximity of emerging LDs within adipocytes with surrounding ER sheaths is schematically depicted in **Fig. 14c**. Perilipin binds to the surface of LDs due to the alternating hydrophobic – hydrophilic short sequence domains and then vimentin wraps these LD-PLIN complexes tightly in cage-like spherical structures, followed by a surrounding of multiple concentric layers of ER cisternae. This arrangement is delineated in a simplified bar-illustrated transversal section at the bottom of the scheme. This latter schematic scenario is illustrated in **Fig. 14c** by a corresponding enlarged EM picture, showing sheaths of ER cisternae covering one droplet. Seen in this particular example are six ER layers, regularly spaced dots of transversally sectioned vimentin IFs (approx. 25 nm distance “high density spacing”) and a small rim of proteins directly contacting the LD surface.

### Various stages of LD formation seen in early adipogenesis

**Figure 15:**
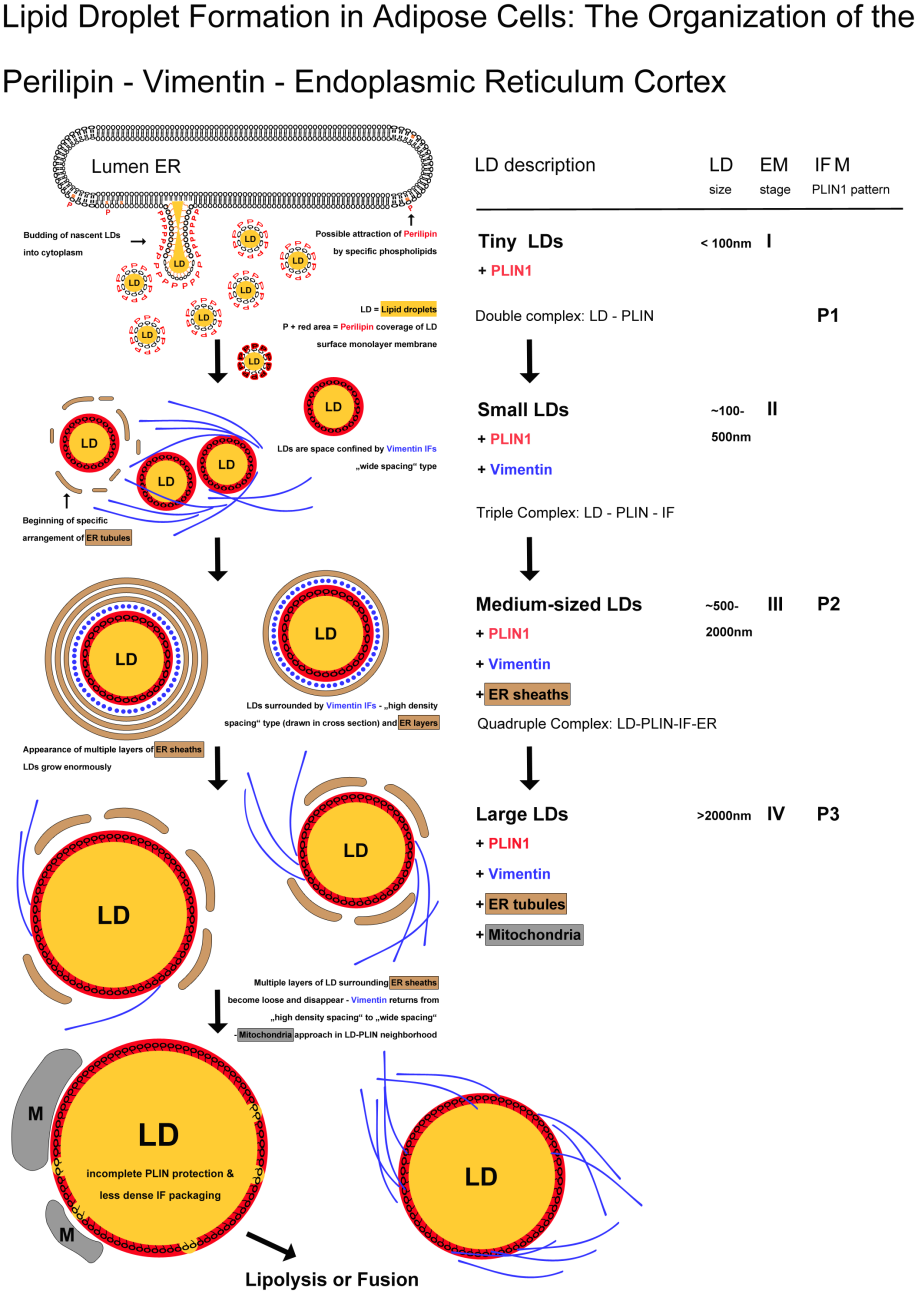
The pathway of LD formation in adipose cells featured by perilipin and vimentin localization. Delineated stages of the endogenous LD-formation are schematically presented with additional brief descriptions of individual steps and involved proteins and organelles, respectively. Sequential events in recruiting and combining proteins and organelles are drawn. The LDs exhibit phospholipid monolayer membranes with perilipin densely covering their surfaces. Perilipin attracts vimentin which further in turn seems to attract a specific ER tubular system (see results for more details). Outlined are double (LD-PLIN), triple (LD-PLIN-IF) and quadruple (LD-PLIN-IF-ER) complexes together with increasing sizes of LDs. Finally, the filament and ER parts of these complexes were relieved by so far unknown influences. In the last stages of differentiation, after the disappearance of the ER sheaths and vimentin, some mitochondria are seen to approach the perilipin-covered large LDs. EM, electron microscopy; for EM stages II-IV, see **Figs. 9** **–** **11** (EM stage I with the direct budding of tiny LDs from ER is rather difficult to see and therefore is drawn here as a suggested mechanism); for correlated different patterns of perilipin in immunofluorescence microscopy (IFM), see **Fig. 7**. ER, endoplasmic reticulum; M, mitochondria; PLIN, perilipin; LD, lipid droplet; IF, intermediated-sized filaments, i.e. vimentin in adipocytes; O =  and O∼, membrane phospholipids and cholesterol in ER bilayer membrane.

Delineated stages of the endogenous LD-formation pathway in adipose cells are schematically presented in **Fig. 15**. The summary of our data gives additional brief descriptions and interpretations of distinguishable steps and on the proteins and organelles involved. The first step with the accumulation of lipids between the two leaflets of the ER membrane and the budding of tiny LDs (stage EM-I) into the cytoplasm follows the prevailing hypothesis on the origin of endogenous LD formation, see e.g. [1], [18], [19], [24], [25]. For next steps, we found ordered sequential events in recruiting and combining of the involved organelles: LDs, ER and mitochondria. Double (LD-PLIN), triple (LD-PLIN-IF) and finally quadruple (LD-PLIN-IF-ER) complexes are outlined together with increasing sizes of LDs during differentiation. Finally, the IF and ER parts of these complexes were released by so far unknown influences and the adipocytes contain mostly large, fully grown LDs with phospholipid monolayer membranes and perilipin densely covering their surfaces. Only in these later stages of adipose differentiation, the perilipin-covered large LDs – without ER sheaths and vimentin – are approached by mitochondria. Then either lipolysis or fusion of LDs might follow.

## Discussion

By initially studying the mere factors which regulate LDs in general and the biogenesis of LDs in particular in human adipose cells, we realized that adipogenic conversion protocols, although suitable to yield high amounts of large LDs, were not favorable for the study of LD biogenesis, including the very small emerging LDs. The usual protocols of shifting the nascent LDs almost completely towards larger coalesced LDs seemed not appropriate when one intended to get information on initial events of LD formation. Therefore our focus was not only on the top-layer fractions, containing mainly large LDs within the currently used density gradient separations, but rather we switched to fractions with higher density containing tiny LDs with less lipid content. We shortened the adipogenic conversion stimulation protocols and enriched small LDs from these fractions by immunoprecipitation (IP) using antibodies highly specific for PLIN proteins. As certain lipophilic dyes often stain only medium-sized and large LDs (see e.g. [9]), and because they are not well-defined at the molecular level, we used well characterized PLIN antibodies. Important and almost imperative was the use of high quality electron microscopy (EM) including immunoelectron microscopy (IEM).

Of additional importance is to keep in mind that commonly applied cell disruption and LD isolation methods favor ”stickiness” and “smearing” effects and thus are very likely prone for protein contamination [2], [13]. Unspecific contamination is, in fact, almost unavoidable. Many hits of proteomic LD experiments given in literature for the most remained not confirmed by additional methods and should therefore be considered with precaution.

Conversion in most of the described low adipose cell passages (passages 2-5 of “Poietic cells”) started more or less synchronically and LD sizes did not differ greatly. Loo et al. [26] reported on particular stages of adipogenesis, on asynchronous differentiation and on subpopulations, revealing quite different amounts of marker proteins. We also observed differences with higher cell passages, more and more cells responded only slowly or not at all to the stimulation additives. We therefore stopped working with higher cell passages and always used fresh, low passages of adipose cells.

### Endogenous- and exogenous-derived LDs – not all LDs in a cell are identical

Perilipin is endogenously newly synthesized and expressed upon adipose conversion. This protein binds strongly to the surface of LDs, thereby preventing e.g. lipase activities. It is thought that perilipin replaces completely other PLIN family proteins originally present in preadipocytes and associated with smaller LDs [18], [27]. In differentiating adipocytes, the LDs contain mainly triglycerides (TAG) and cholesterol-esters (CE). These hydrophobic substances are supposed to be generated within the bilayer of the ER and by the LD biogenesis process stimulated upon AIM treatment. The arising LDs - in contrast to other cell organelles – possess a monolayer membrane ([18], [19], [24], [28], [29]; for recent overview see [30]). We challenged the endogenously generated LDs with oleic acid (OA) supplied to the culture medium, generating thereby different LDs, some with monolayer (by AIM) and others with bilayer membranes (by OA). A report on “two lipid storing organelles” – one by LD formation within the bilayer of ER and a second one by lipid endocytosis and lipid storage at the plasma membrane caveolae [31] - gave us the idea for such double treatment. In principle the monolayer vs. bilayer membrane situation was outlined also in a scheme shown by Fujimoto et al. [1]. These authors questioned how LDs can fuse with endocytosed caveolin-vesicles. By double stimulation with AIM and OA treatment of preadipocytes, we received a variety of diverse LDs which had quite different sets of PLIN proteins binding to LD surfaces. Single-color-stained LDs, mosaic, heterogeneously mixed and merged LDs positive for PLIN proteins in various sizes were detectable. Many different types of LDs in single cells could be generated by combining specific **“endogenous”** adipogenic stimulation and **“exogenous”** OA-treatment. We noticed, after AIM conversion and upon the uptake of OA, a rapid increase in expression of adipophilin, TIP47 and S3-12. Obviously these latter proteins were independently involved in the endocytotic uptake of hydrophobic substances, in contrast to the endogenously derived perilipin expression at the ER. These results are in conflict with current models of LD maturation in adipocytes, where TIP47, S3-12 and adipophilin are thought to be replaced continuously by newly expressed perilipin ([27]; for “EPAT exchangeable model” see [32]; for TIP47 (PLIN3) at ER see [33]). Preliminary experiments of *in vitro* binding using recombinant PLIN proteins to “Membrane Lipid Strips” (Echelon Biosciences), testing spotted lipid substances, revealed different sets of binding pattern for perilipin (PLIN1), adipophilin (PLIN2), TIP47 (PLIN3) and MLDP (PLIN5) respectively, suggesting that each PLIN protein is specialized to different sets of lipid material (H.Heid, unpublished). This confirms the work of Hsieh [23] on LD sorting and on specific binding with preferences of individual PLIN proteins to diverse hydrophobic components, mainly either TAG- or mainly CE-containing droplets. Heterogeneities and diversities of LD-binding proteins at the surface of LDs in single cells were also demonstrated e.g. by Ozaki et al. [34] and Thiele & Spandl [35].

The mechanism of potential merging of different kind of LDs, the endogenously derived ones with monolayer biomembranes and the exogenously derived ones with bilayer biomembranes is unknown. As already mentioned, Fujimoto et al. [1] emphasized the two different types of LDs, but these authors did not describe that the caveolin-vesicles can also be coated by PLIN proteins (see e.g. [36] [37]). Interestingly, by immunoprecipitations (IPs) using LD fractions, we detected proteins that are known to play central roles in receptor-mediated endocytosis and vesicle transport: clathrin heavy chain 1, AP2 adaptor protein, CALM - a clathrin assembly protein - and caveolin-1 (cp. similar IP results with epithelium-derived cells [13]). Clathrin heavy chain 22 (CHC22) is reported to be highly expressed in muscle and fat and to be associated with AP1 and AP3 adaptors - but exclusively not with the endocytic AP2 complex ([38]). Yet with our fat cells, we now precipitated clathrin and - just in opposition to this reference - AP2 adaptor protein (**,b**). In this context, several reports are of interest which were published in recent years dealing with impaired adipogenesis and lipodystrophies with involvement of affected genes for caveolin-1, cavin-1 and perilipin (for review see [39]). Whether hydrophobic substances like OA are delivered eventually via a late endosome-endolysosomal pathway or by an endosome-Golgi pathway with involvement of COPI and COPII to the ER [40]–[42] and how OA-containing droplets combine with endogenous TAG- and CE-containing droplets, remains to be elucidated.

A summary of our various treatments of adipose cell is given in **Fig. 3**. The conversion of preadipocytes resulted in the expression and changes of individual PLIN proteins. For further investigations, we worked preferentially with cells designated “Adipocytes” and “OA-Adipocytes”.

### Perilipin and vimentin – a special partnership for hydrophobic cases

Using gradient separations and IPs, we found direct interaction of vimentin with perilipin and hence perilipin as the major linker protein of LDs and the intermediate-sized filaments (IFs) network in adipocytes. The association of vimentin with LDs and with lipids was already described in literature [15] but perilipin was not discovered at the time of these publications and therefore could not be identified as a missing link. The specific binding of these two proteins - perilipin and vimentin – shown here for the first time, shows some extraordinary features. Perilipin tends to accumulate with LDs in top layers of separations whereas vimentin is rather insoluble in physiological buffers and is preferentially found in sediments. Therefore using normal protein isolation methods, it is rather unlikely that one finds the two proteins together in one fraction, because each of these proteins affords a quite different isolation method. The biochemical data and the IFM pictures gave us first clues of a direct protein-protein interaction and these data were finally confirmed with IEM. We conclude that the amphiphilic protein perilipin acts as a mediator, to combine LDs with the IF network. Both proteins together, as tightly sealed complex, are assumed to protect LDs in combination, e.g. against lipase activity.

### The LD-PLIN-vimentin model

After having established the localization data, we speculated how binding between perilipin and vimentin could function. Perilipin is expressed preferentially in fat cells and its amino acid sequence does not contain the reported 4 helical domains at the C-terminus when compared with the other PLIN proteins [43]. All other PLIN proteins are expressed more pervasively in many cell types. The helical domains of the PLIN proteins were proposed to be responsible for the interaction with cytokeratins in epithelium-derived cells [13]. Instead of helical domains, perilipin exhibits - as the only PLIN family member - a conspicuous glutamic acid-rich (**E-rich**) domain. In adipocytes, we are dealing only with vimentin as single intermediate-sized filament. Therefore, we checked both sequences for motifs which could be candidate regions for specific binding and found a possible antipode to the acidic E-rich domain of perilipin, the basic arginine-rich (R-rich) region within the head sequence of vimentin (Fig. 14). Regarding the many annotated serine phosphorylation sites (“hot spots”) within the head region of vimentin, situated between the arginines of the R-rich region, it is tempting to speculate that the tight binding between perilipin and vimentin based on opposite charges and ionic forces is regulated by phosphorylation (i.e. phosphatases, kinases). In literature e.g., increasing phosphorylation of perilipin is reported to stimulate lipolysis [44] and phosphorylation of vimentin is leading ultimately to accelerated cholesterol transport in adrenal steroidogenesis [45]. Niemann-Pick type C disease – a severe neurodegenerative disorder with massive accumulation of cholesterol, fatty acids and other hydrophobic substances – showed vimentin hypophosphorylation leading to lipid transport defects [46].

Disruption of the vimentin filament system in 3T3-L1 mouse adipocytes by injection of antibodies was leading to LD accumulation [47]. These and also other additional experiments for further testing the proposed PLIN-vimentin binding could be performed now using e.g. perilipin-knockout mice, vimentin-knockout mice and e.g. SW-13 adrenal tumor cells that contain or lack vimentin filaments [48]–[52].

### Different perilipin staining patterns and multiple rings of ER cisternae

During adipose conversion of human adipose cells, we found, with antibodies directed against the N-terminus of perilipin, a normal staining pattern. However, when using antibodies against the C-terminus of perilipin, we recognized additional new and so far not described patterns. In the latter case, we could distinguish 3 distinct perilipin patterns (“P1–P3”). We conclude that not every antibody against perilipin shows the entire picture and tentatively interpret the very fine, punctate perilipin pattern associated alongside vimentin filaments in the perinuclear region (“P1”) as position of LD biosynthesis at the ER. The differences seen with the two perilipin antibodies might be due to a different accessibility of epitopes in the forming of LD-protein complexes.

In EM, we found specific arrangements and changes of vimentin filaments (“wide spacing”) in nascent lipid globules. In advanced differentiated cells, the LD complexes are surrounded by a monolayer of regular vimentin IFs (“high density spacing”) that in turn is closely ensheathed by multiple special ER cisternae. One layer of ER cisternae is often seen wrapping partly, or in some cases, completely around LDs [15], [16], [20], [24], [34], [42], [53]. But, to our knowledge higher numbers of those ER rings have not been reported in differentiating adipocytes. Whether the “multiple parallel lines” or the “concentric lipid ester layers” shown by cryoelectron microscopy [2], [29] are related structural components remains unclear. Other reports do not show multiple rings of cisternae, possibly be due to the longer conversion protocols used in contrast to our short conversion. At present we saw similar EM pictures with multiple layers of surrounding LDs cisternae in Leydig cells, sertoli cells and adrenal cells seen in the atlas collection of Don Fawcett [54]. However, in these hormone-producing cells, the ER sheaths are seen fenestrated, whereas in the fat cells shown here, the ER structures are clearly non-fenestrated. Whether these structural differences originate from and depend on the production of different hydrophobic substances, remains to be investigated.

### Perilipin and vimentin in adipogenesis

In general LDs consist of a hydrophobic core of triglycerides and sterol esters, having only one leaflet derived from the endoplasmic reticulum membrane. Our data on endogenously derived LDs are summarized within the scheme shown in **Fig. 15**, giving a possible pathway of LD formation. Delineated are stages of early adipose cell differentiation with brief descriptions and interpretations seen mainly as results of our data based on EM and IEM. The EM pictures suggest the existence of at least three different types of LDs in differentiating adipocytes: **(1)** very small LDs, often seen associated with vimentin filaments, **(2)** medium-sized LDs tightly enwrapped, cage-like by vimentin filaments and surrounded by multiple ER cisternae and **(3)** bigger LDs only partly associated with IFs and single ER cisternae. Whether these different types of LDs seen in EM correlate with the 3 perilipin patterns seen in IFM (“**P1-P3”**) remains to be established.

We describe endogenously derived LDs in adipogenesis which in ordered sequential events recruit and combine with specific proteins and organelles. Outlined are double (LD-PLIN), triple (LD-PLIN-IF) and finally quadruple (LD-PLIN-IF-ER) complexes together with increasing sizes of LDs. Taken together, with the data on exogenously-derived LDs, new possibilities and experiments to obtain additional insights on the complexity of LD formation for a new platform and discussion are presented.

## Supporting Information

Figure S1 **Conventional adipogenic stimulation of preadipocytes in culture cells.** A schematic overview including a short description of treatment of adipose cells and perilipin (PLIN) proteins involved in this differentiation process is given. For conversion into mature fat cells elongated preadipocytes, containing long dendrites and small lipid droplets (LDs; surface staining for Adipophilin, TIP47 and S3-12 in green) are treated with adipocyte differentiation medium (also described as adipocyte induction medium, AIM). Newly emerging medium-sized and large LDs, endogenously generated are stained for perilipin (red). In differentiated cells adipophilin, TIP47 and S3-12 expression is mostly reduced and the corresponding small LDs are barely visible at later stages of conversion. Scheme modified from textbook [55].
(TIF)

Figure S2 **Laser scanning immunofluorescence microscopy showing lipid droplet (LD) adipophilin-labeling in non-stimulated human preadipocytes.** The adipophilin monoclonal antibody (mab) reveals many small LDs (green) distributed all over the cytoplasm - including localization in long dendrites (examples marked by arrowheads). Nuclear staining was with DAPI (blue). The corresponding digital image correlation (DIC) picture is shown in combination with the picture of the immunofluorescence micrograph. Bar: 20 µm.
(TIF)

Figure S3 **Proteomic analysis of immunoprecipitated density gradient fractions using AIM-stimulated human preadipocytes.** (**a**) Silver-stained SDS-acrylamide gel separation of proteins obtained by specific immunoprecipitations (IPs) is shown. Aliquots of gradient fraction **LD2** (cp. **Fig. 4**) utilized for IPs with various monoclonal antibodies are shown. L: Used sample lysate for IPs. M: Marker proteins. Peri-IP: obtained with mab Peri112.17. Vim-IP: obtained with mab VIM 3B4. AP-IP: obtained with mab AP125. VE-IP: Control IP obtained with mab VE-Cadherin. **(-)**: Control obtained without specific 1^st^ mab. At the left margin the positions of molecular weight (mw) markers and at the right side the position of co-precipitated immunoglobulin bands (asterisks) are given. (**b,c**) Individual areas of gel lanes used for tryptic digests followed by mass spectrometry (MS) analysis are indicated by rectangles and numbers 1-13 respectively. (**b**) IP employing perilipin antibody and detection of known LD-binding proteins received by analyzing the corresponding complete gel lane by MS. (**c**) IP employing vimentin antibody and detection of known LD-binding proteins received by analyzing the corresponding complete lane by MS. Note: The precipitates of mabs Peri112.17 and VIM 3B4 resulted in very similar proteomic “hits”, e.g. besides perilipin and vimentin, the known LD-binding proteins S3-12 (within various mw regions), TIP47, 100 kD coactivator protein, Rab18, respectively. For detailed lists of MS results see **Tables S2a,b**.
(TIF)

Figure S4 **Immunoelectron microscopic localization of perilipin in briefly AIM-stimulated and OA-treated human preadipocytes.** By additional treatment with OA, some supposedly exogenous-derived LDs (labeled **LD-exo**) revealing no perilipin specific staining can be detected. These LDs are found in the midst of many endogenously-derived mab perilipin-positive LDs which in turn are triggered by AIM stimulation. All LDs are seen closely associated and anchored with IF bundles. Bars: 0.50 µm.
(TIF)

Table S1 **Antibodies used.** Antibody designation, animal source and amino acid (aa) positions of used peptides of PLIN proteins for immunization are given. Polypeptides were synthesized (PSL, Heidelberg, Germany) and conjugated to keyhole limpet hemocyanin (KLH) to trigger and enhance immunoreaction. NT  =  N-terminal; CT  =  C-terminal; h  =  human; m  =  mouse; gp  =  guinea pig; mab  =  monoclonal antibody; pab  =  polyclonal antibody; aa-Position  =  amino acid positions of peptides selected from human protein sequences used for generation of antibodies. Monoclonal antibodies specific for adipophilin and perilipin were generated by the Helmholtz Group for Cell Biology (German Cancer Research Center) using KLH-coupled polypeptides for immunization and BALB/c mice. The mab specific for vimentin (clone VIM 3B4) was generated by PROGEN Biotechnik, Heidelberg, Germany, using native vimentin isolated from bovine lens (bVimentin). The mab specific for VE-Cadherin (clone BV9), used as a control antibody in immunoprecipitations (IPs), was a generous gift of E. Dejana, University of Milan, Italy. Note, in many experiments we used in parallel for controlling and confirmation different epitope-specific antibodies of individual PLIN proteins. In certain cases these experiments led to the recognition of different staining patterns and/or accessibilities of individual PLIN proteins (e.g. differences were seen using N-terminal vs. C-terminal specific perilipin antibodies, **Figs. 6** **,** **7**; cp. also different staining seen with two TIP47 specific antibodies, shown in **Fig. 2f** by Heid et al., [13]). The antibodies are commercially available from PROGEN. For extended list of antibodies generated against LD-binding proteins used in control experiments (not shown) see **Table S1** in Heid et al., [13].
(PDF)

Table S2 **Detailed proteomic lists of immunoprecipitated density gradient fractions using AIM-stimulated human preadipocytes**. The complete silver-stained gel lanes of the perilipin (Table 2a) and vimentin (Table 2b) IPs were used for MS analysis respectively and results listed (cp. Fig. S3). Explanations regarding sample numbers, data base accession numbers of identified human proteins, brief protein descriptions, scores, predicted molecular weights and number of hits are given within the lists. Color codes are assigned for designation of distinct groups of hits and preliminary grouping. Note: The red color code highlights known LD-binding proteins. PLIN proteins Perilipin, S3-12 and TIP47 are detected with expected molecular weight but can also be found in samples with lower or higher molecular weights, presumably due to degradation or crosslinking with partner proteins. Within the samples vimentin is also identified with very high scores. Proteins involved in fatty acid, steroid- and lipid pathways are marked in green color code, and proteins mainly localized at the endoplasmatic reticulum (ER), in vesicles or involved in endocytosis are colored yellow. Note in addition: All identified IgGs, serum albumin, epidermal keratins and hits with very low scores were excluded. Many of the given proteins are assigned by data base numbers only or cannot be assigned exactly with the given information obtained from data bases (without color). Therefore, many of these assignments are preliminary and not confirmed.
(PDF)
