# Supplementary figures and images for: On the Formation of Lipid Droplets in Human Adipocytes: The Organization of the Perilipin–Vimentin Cortex
Source: PLoS One. 2014 Feb 28;9(2):e90386. doi: 10.1371/journal.pone.0090386 (PMC3938729; doi:10.1371/journal.pone.0090386)

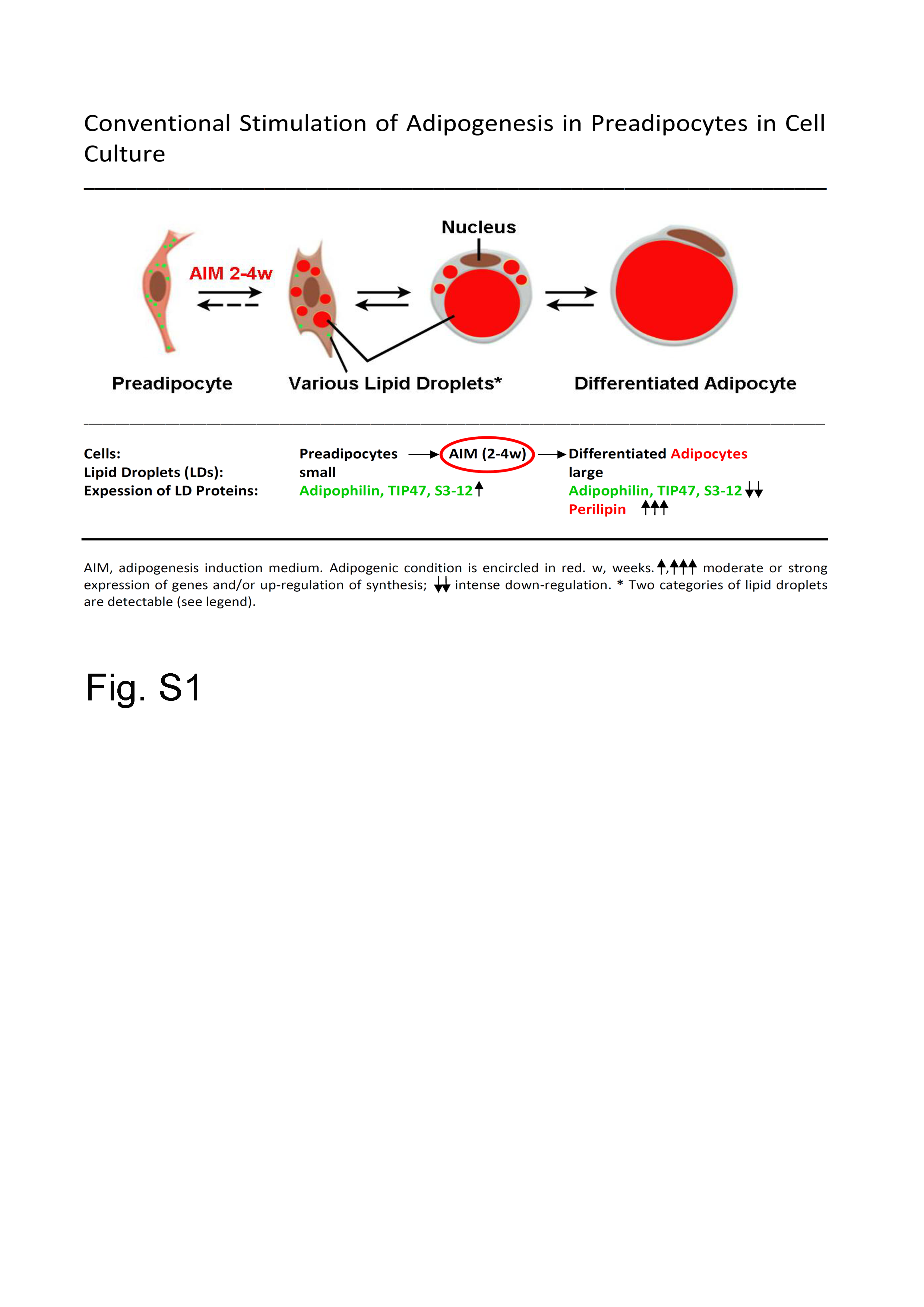

Supplement: Figure S1 — Conventional adipogenic stimulation of preadipocytes in culture cells. A schematic overview including a short description of treatment of adipose cells and perilipin (PLIN) proteins involved in this differentiation process is given. For conversion into mature fat cells elongated preadipocytes, containing long dendrites and small lipid droplets (LDs; surface staining for Adipophilin, TIP47 and S3-12 in green) are treated with adipocyte differentiation medium (also described as adipocyte induction medium, AIM). Newly emerging medium-sized and large LDs, endogenously generated are stained for perilipin (red). In differentiated cells adipophilin, TIP47 and S3-12 expression is mostly reduced and the corresponding small LDs are barely visible at later stages of conversion. Scheme modified from textbook [55]. (TIF) [file pone.0090386.s001.tif]

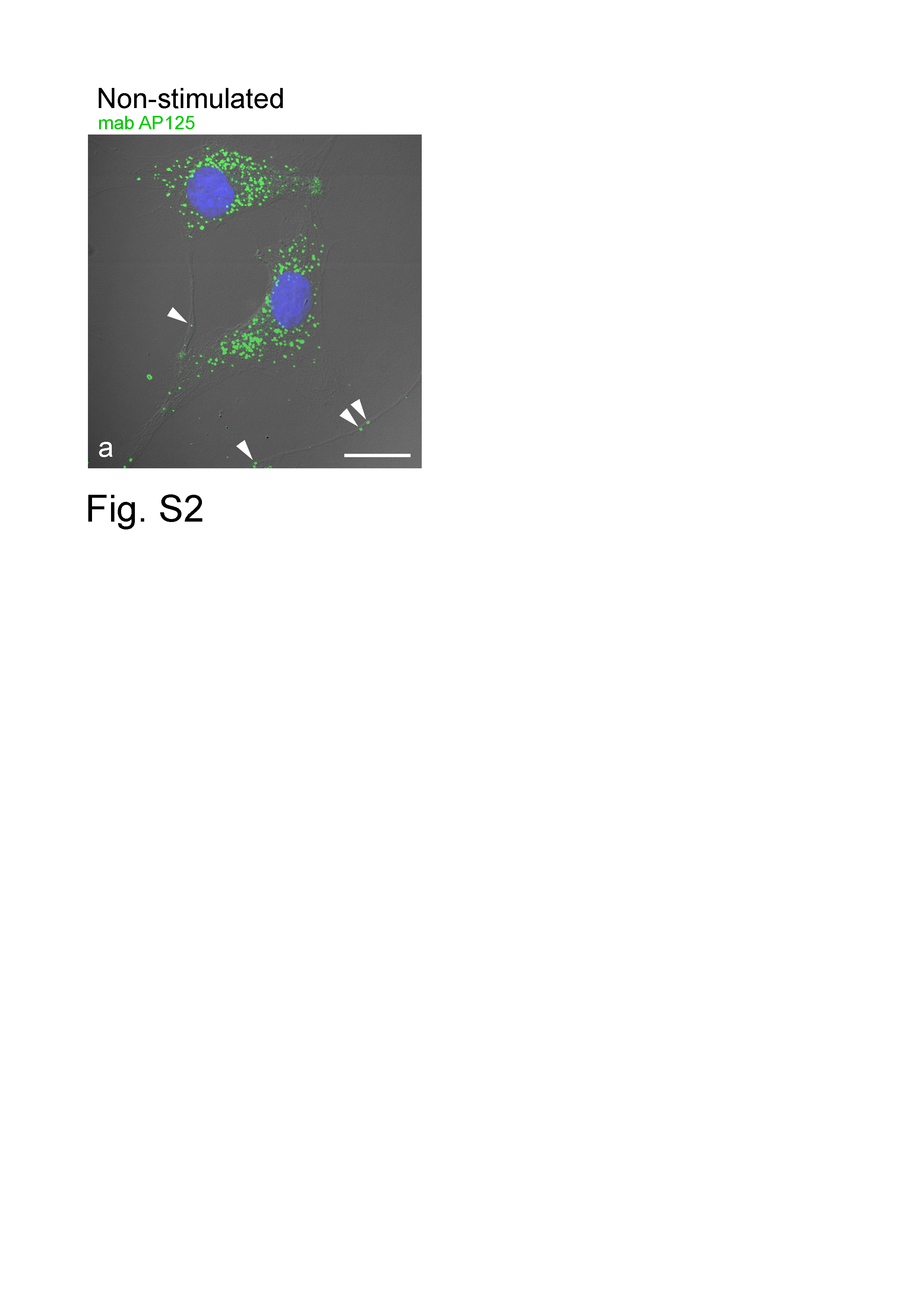

Supplement: Figure S2 — Laser scanning immunofluorescence microscopy showing lipid droplet (LD) adipophilin-labeling in non-stimulated human preadipocytes. The adipophilin monoclonal antibody (mab) reveals many small LDs (green) distributed all over the cytoplasm - including localization in long dendrites (examples marked by arrowheads). Nuclear staining was with DAPI (blue). The corresponding digital image correlation (DIC) picture is shown in combination with the picture of the immunofluorescence micrograph. Bar: 20 µm. (TIF) [file pone.0090386.s002.tif]

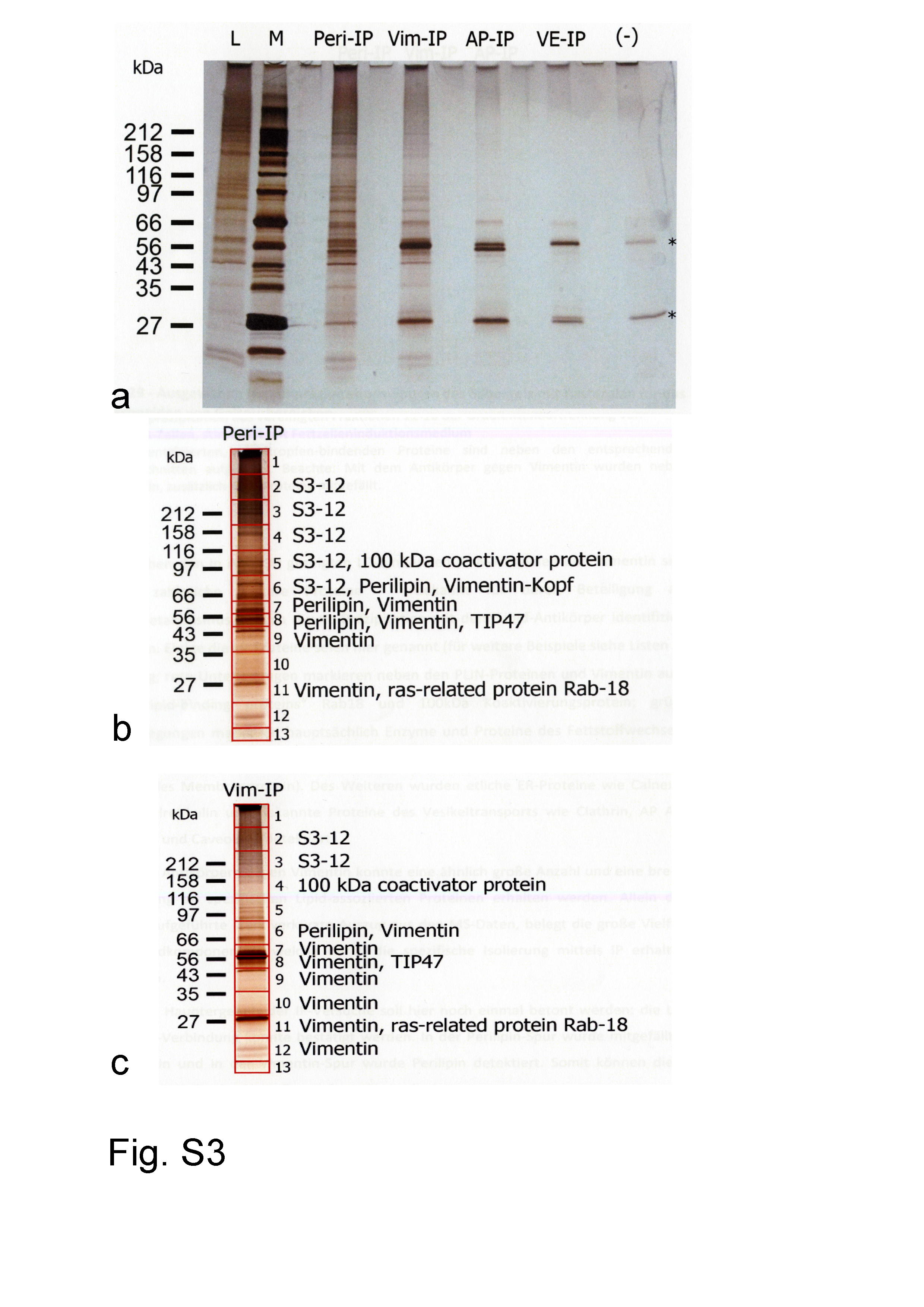

Supplement: Figure S3 — Proteomic analysis of immunoprecipitated density gradient fractions using AIM-stimulated human preadipocytes. (a) Silver-stained SDS-acrylamide gel separation of proteins obtained by specific immunoprecipitations (IPs) is shown. Aliquots of gradient fraction LD2 (cp. Fig. 4 ) utilized for IPs with various monoclonal antibodies are shown. L: Used sample lysate for IPs. M: Marker proteins. Peri-IP: obtained with mab Peri112.17. Vim-IP: obtained with mab VIM 3B4. AP-IP: obtained with mab AP125. VE-IP: Control IP obtained with mab VE-Cadherin. (-): Control obtained without specific 1st mab. At the left margin the positions of molecular weight (mw) markers and at the right side the position of co-precipitated immunoglobulin bands (asterisks) are given. (b,c) Individual areas of gel lanes used for tryptic digests followed by mass spectrometry (MS) analysis are indicated by rectangles and numbers 1-13 respectively. (b) IP employing perilipin antibody and detection of known LD-binding proteins received by analyzing the corresponding complete gel lane by MS. (c) IP employing vimentin antibody and detection of known LD-binding proteins received by analyzing the corresponding complete lane by MS. Note: The precipitates of mabs Peri112.17 and VIM 3B4 resulted in very similar proteomic “hits”, e.g. besides perilipin and vimentin, the known LD-binding proteins S3-12 (within various mw regions), TIP47, 100 kD coactivator protein, Rab18, respectively. For detailed lists of MS results see Tables S2a,b. (TIF) [file pone.0090386.s003.tif]

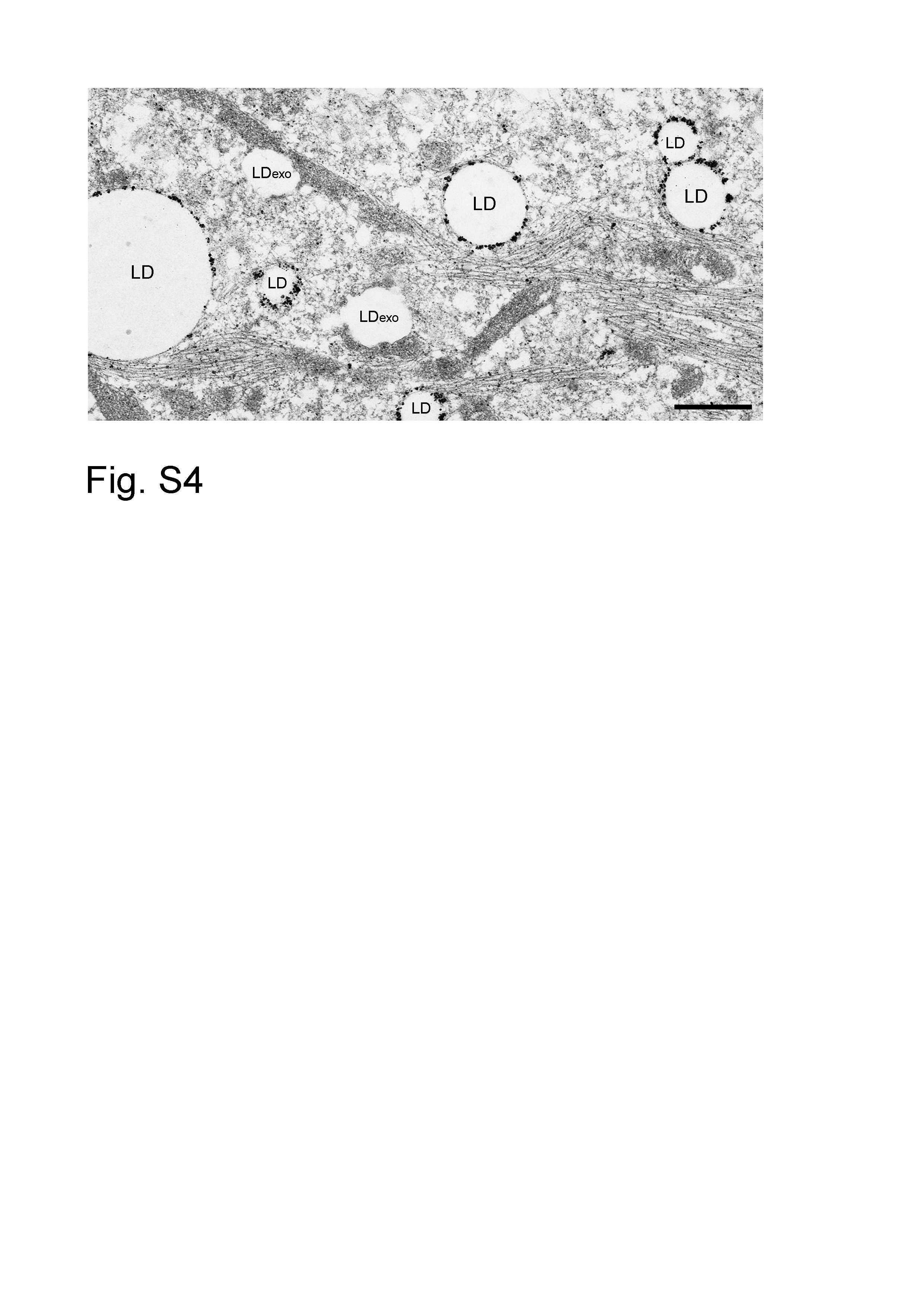

Supplement: Figure S4 — Immunoelectron microscopic localization of perilipin in briefly AIM-stimulated and OA-treated human preadipocytes. By additional treatment with OA, some supposedly exogenous-derived LDs (labeled LD-exo) revealing no perilipin specific staining can be detected. These LDs are found in the midst of many endogenously-derived mab perilipin-positive LDs which in turn are triggered by AIM stimulation. All LDs are seen closely associated and anchored with IF bundles. Bars: 0.50 µm. (TIF) [file pone.0090386.s004.tif]
