# Supplementary material for: On the Formation of Lipid Droplets in Human Adipocytes: The Organization of the Perilipin–Vimentin Cortex
Source: PLoS One. 2014 Feb 28;9(2):e90386. doi: 10.1371/journal.pone.0090386 (PMC3938729; doi:10.1371/journal.pone.0090386)
Supplement: Table S1 — Antibodies used. Antibody designation, animal source and amino acid (aa) positions of used peptides of PLIN proteins for immunization are given. Polypeptides were synthesized (PSL, Heidelberg, Germany) and conjugated to keyhole limpet hemocyanin (KLH) to trigger and enhance immunoreaction. NT = N-terminal; CT = C-terminal; h = human; m = mouse; gp = guinea pig; mab = monoclonal antibody; pab = polyclonal antibody; aa-Position = amino acid positions of peptides selected from human protein sequences used for generation of antibodies. Monoclonal antibodies specific for adipophilin and perilipin were generated by the Helmholtz Group for Cell Biology (German Cancer Research Center) using KLH-coupled polypeptides for immunization and BALB/c mice. The mab specific for vimentin (clone VIM 3B4) was generated by PROGEN Biotechnik, Heidelberg, Germany, using native vimentin isolated from bovine lens (bVimentin). The mab specific for VE-Cadherin (clone BV9), used as a control antibody in immunoprecipitations (IPs), was a generous gift of E. Dejana, University of Milan, Italy. Note, in many experiments we used in parallel for controlling and confirmation different epitope-specific antibodies of individual PLIN proteins. In certain cases these experiments led to the recognition of different staining patterns and/or accessibilities of individual PLIN proteins (e.g. differences were seen using N-terminal vs. C-terminal specific perilipin antibodies, Figs. 6 , 7 ; cp. also different staining seen with two TIP47 specific antibodies, shown in Fig. 2f by Heid et al., [13]). The antibodies are commercially available from PROGEN. For extended list of antibodies generated against LD-binding proteins used in control experiments (not shown) see Table S1 in Heid et al., [13]. (PDF) [file pone.0090386.s005.pdf]

Antibodies used

Antibody designation	Source	aa-Position
**Perilipin**		
mab Peri 112.17.1	m	1-20
pab Peri-h+NT	gp	1-20
pab Peri-hCT	gp	507-519
**Adipophilin**		
mab AP125	m	6-28
pab Adipo-hCT	gp	423-437
**TIP47**		
pab TIP47-hCT	gp	420-431
**S3-12**		
pab S3-12-hNT	gp	1-15
pab S3-12-hCT	gp	1385-1400
**Vimentin**		
mab VIM 3B4	m	bVimentin
**VE-Cadherin**		
mab BV9	m	

Table S1
