# Supplementary material for: On the Formation of Lipid Droplets in Human Adipocytes: The Organization of the Perilipin–Vimentin Cortex
Source: PLoS One. 2014 Feb 28;9(2):e90386. doi: 10.1371/journal.pone.0090386 (PMC3938729; doi:10.1371/journal.pone.0090386)
Supplement: Table S2 — Detailed proteomic lists of immunoprecipitated density gradient fractions using AIM-stimulated human preadipocytes. The complete silver-stained gel lanes of the perilipin (Table 2a) and vimentin (Table 2b) IPs were used for MS analysis respectively and results listed (cp. Fig. S3). Explanations regarding sample numbers, data base accession numbers of identified human proteins, brief protein descriptions, scores, predicted molecular weights and number of hits are given within the lists. Color codes are assigned for designation of distinct groups of hits and preliminary grouping. Note: The red color code highlights known LD-binding proteins. PLIN proteins Perilipin, S3-12 and TIP47 are detected with expected molecular weight but can also be found in samples with lower or higher molecular weights, presumably due to degradation or crosslinking with partner proteins. Within the samples vimentin is also identified with very high scores. Proteins involved in fatty acid, steroid- and lipid pathways are marked in green color code, and proteins mainly localized at the endoplasmatic reticulum (ER), in vesicles or involved in endocytosis are colored yellow. Note in addition: All identified IgGs, serum albumin, epidermal keratins and hits with very low scores were excluded. Many of the given proteins are assigned by data base numbers only or cannot be assigned exactly with the given information obtained from data bases (without color). Therefore, many of these assignments are preliminary and not confirmed. (PDF) [file pone.0090386.s006.pdf]

Table S2a

IP : Poietics AIM **Peri 112.17.1 mab**

A1

Hit No.	Accession No.	Protein Description	Score	Mass [Da]	Matches	Coverage [%]	
12	gi|179212	Na+ K+ ATPase alpha subunit, partial	274	82706	10	8,8	integral plasma membrane protein,
16	gi|181184	stomatin peptide	118	31860	3	11,1	lipid raft-associated integral membrane protein
17	gi|31092	unnamed protein product	110	50437	5	5,6	elongation factor
19	gi|4506675	dolichyl-diphosphooligosaccharide--protein glycosyltransferase subunit 1 precursor	102	68641	4	6,1	sugar transfer from dolichol-phosphate-sugars occurs exclusively on the luminal side of the ER
20	gi|2414516	surface 4 integral membrane protein	101	28724	3	12,2	
24	gi|4757756	annexin A2 isoform 2	86	38808	2	6,8	calcium-dependent phospholipid-binding protein

A2

Hit No.	Accession No.	Protein Description	Score	Mass [Da]	Matches	Coverage [%]	
1	gi|61743954	neuroblast differentiation-associated protein AHNAK isoform 1	1591	629213	70	9	AHNAK binds annexin and works together in the developement of intracellular membranes
2	gi|15620821	KIAA1881 protein	976	133891	27	17,8	S3-12, lipid droplet-binding
4	gi|219841772	COL6A3 protein	754	279629	20	8,4	
8	gi|41584442	fatty acid synthase	243	275900	8	3	multi-enzyme, catalyzes the synthesis of palmitate from acetyl-CoA & malonyl-CoA to long-chain saturated fatty acids
11	gi|3299885	ES/130	187	109019	5	6,9	ribosome receptor
12	gi|34339	LDL-receptor related precursor (AA -19 to 4525)	181	523119	5	1,2	central role in mammalian cholesterol metabolism; the receptor protein mammalian cholesterol metabolism
15	gi|181184	stomatin peptide	147	31860	4	9,4	lipid raft-associated integral membrane protein
18	gi|4502119	membrane primary amine oxidase	99	85138	3	2,6	oxidative conversion of amines to aldehydes, major protein on the adipocyte plasma membrane
25	gi|16945323	CD92 protein	70	74473	3	4	plasma-membrane choline transporter
26	gi|4506675	dolichyl-diphosphooligosaccharide--protein glycosyltransferase subunit 1 precursor	69	68641	2	3,8	sugar transfer from dolichol-phosphate-sugars occurs exclusively on the luminal side of the ER

A3

Hit No.	Accession No.	Protein Description	Score	Mass [Da]	Matches	Coverage [%]	
1	gi|119591516	collagen, type VI, alpha 3, isoform CRA_h	2249	323451	85	19,7	
2	gi|61743954	neuroblast differentiation-associated protein AHNAK isoform 1	1435	629213	61	9,3	AHNAK binds annexin and works together in the developement of intracellular membranes
3	gi|12667788	myosin-9	1352	227646	53	18,9	
4	gi|41584442	fatty acid synthase	1270	275900	38	13,5	multi-enzyme, catalyzes the synthesis of palmitate from acetyl-CoA & malonyl-CoA to long-chain saturated fatty acids
5	gi|15620821	KIAA1881 protein	1185	133891	33	20,5	S3-12, lipid droplet-binding
7	gi|7305053	myoferlin isoform a	846	236100	27	10,3	calcium/phospholipid-binding protein
9	gi|34339	LDL-receptor related precursor (AA -19 to 4525)	634	523119	24	4,5	central role in mammalian cholesterol metabolism; the receptor protein mammalian cholesterol metabolism
10	gi|31397	fibronectin precursor	624	260064	23	10	
11	gi|4235275	talin	511	271828	16	7,2	
14	gi|178536	aminopeptidase N precursor (EC 3.4.11.2)	429	109842	13	13,3	
15	gi|189730	platelet-derived growth factor receptor	365	124959	11	11,6	
16	gi|181184	stomatin peptide	333	31860	9	28,5	lipid raft-associated integral membrane protein
17	gi|28243	unnamed protein product	326	283323	11	4,2	actin-binding protein (ABP-280, nonmuscle filamin)
18	gi|3299885	ES/130	317	109019	7	7,9	ribosome receptor
19	gi|1418928	prepro-alpha1(I) collagen	308	139853	12	6,9	
20	gi|4506787	ras GTPase-activating-like protein IQGAP1	281	189761	9	4,4	interacts with components of the cytoskeleton & adhesion molecules to regulate cell morphology & motility
21	gi|4758012	clathrin heavy chain 1	246	193260	7	4,4	involved in endocytosis
22	gi|28336	mutant beta-actin (beta'-actin)	235	42128	12	22,1	
23	gi|35655	unnamed protein product	226	57458	10	18,9	folding in ER
26	gi|2104553	Myosin heavy chain (MHY11) (5'partial)	202	215017	9	2,7	
27	gi|179106	nonerythroid alpha-spectrin	187	284905	4	2,2	
29	gi|930045	alpha-1 (III) collagen	177	96556	7	6,6	
30	gi|5531849	SURF-4 isoform	173	18186	7	40,9	surface integral membrane protein
32	gi|4218955	gamma-filamin	157	291287	4	1,5	actin-binding
34	gi|16945323	CD92 protein	137	74473	5	7,5	plasma-membrane choline transporter
41	gi|4506675	dolichyl-diphosphooligosaccharide--protein glycosyltransferase subunit 1 precursor	91	68641	2	3,8	sugar transfer from dolichol-phosphate-sugars occurs exclusively on the luminal side of the ER
45	gi|4757756	annexin A2 isoform 2	82	38808	4	7,4	calcium-dependent phospholipid-binding protein
46	gi|342350777	Chain A, Human Annexin V With Incorporated Methionine Analogue Azidohomoalanine	73	35811	3	6,9	calcium-dependent phospholipid-binding protein

A4

Hit No.	Accession No.	Protein Description	Score	Mass [Da]	Matches	Coverage [%]	
3	gi|41584442	fatty acid synthase	1161	275900	38	11,8	multi-enzyme, catalyzes the synthesis of palmitate from acetyl-CoA & malonyl-CoA to long-chain saturated fatty acids
8	gi|119589620	hCG1646516	735	133590	22	13,7	S3-12, lipid droplet-binding
9	gi|61743954	neuroblast differentiation-associated protein AHNAK isoform 1	543	629213	21	3	AHNAK binds annexin and works together in the developement of intracellular membranes
10	gi|4507877	vinculin isoform VCL	515	117220	14	12,7	
11	gi|12667788	myosin-9	499	227646	15	7,9	
12	gi|87196339	collagen alpha-1(VI) chain precursor	461	109602	19	13,6	
13	gi|5453832	hypoxia up-regulated protein 1 precursor	433	111494	24	16,6	endoplasmic reticulum (ER) under hypoxic conditions
14	gi|28336	mutant beta-actin (beta'-actin)	431	42128	21	35,7	
15	gi|4504763	integrin alpha-V isoform 1 precursor	431	117062	15	13,9	
16	gi|19743813	integrin beta-1 isoform 1A precursor	410	91664	22	14,8	
18	gi|189730	platelet-derived growth factor receptor	377	124959	11	10,3	
19	gi|7305053	myoferlin isoform a	376	236100	13	5,4	calcium/phospholipid-binding protein

20	gi|458032	aspartyl beta-hydroxylase	362	85958	12	19,4	
21	gi|34339	LDL-receptor related precursor (AA -19 to 4525)	352	523119	14	2,7	central role in mammalian cholesterol metabolism; the receptor protein mammalian cholesterol metabolism
22	gi|1418928	prepro-alpha1(I) collagen	319	139853	8	5,6	
23	gi|41350923	Collagen, type VI, alpha 2	275	109736	9	6,7	
24	gi|4757756	annexin A2 isoform 2	274	38808	9	19,2	calcium-dependent phospholipid-binding protein
26	gi|3882215	KIAA0747 protein	256	120296	9	7,5	synaptotagmin_like
27	gi|4235275	talin	247	271828	6	3	
28	gi|68533085	MYO1C variant protein	246	125833	5	4,6	
29	gi|603074	ATP:citrate lyase	242	121660	6	7,4	cleaves cítryl-CoA (CiCoA) to acetyl-CoA
30	gi|19263767	Similar to cytoskeleton-associated protein 4, partial	225	62194	5	13,6	
31	gi|31438	unnamed protein product	220	115577	5	4,7	integrin
32	gi|4758012	clathrin heavy chain 1	216	193260	8	4,2	involved in endocytosis
33	gi|35655	unnamed protein product	215	57458	8	13,6	oxidative protein folding in the ER
34	gi|1418930	prepro-alpha2(I) collagen	211	129772	6	6	
35	gi|181184	stomatin peptide	209	31860	6	19,1	lipid raft-associated integral membrane protein
38	gi|296164	156 kDa Protein	185	156282	4	3,9	kinectin, perinuclear and ER

A5

Hit No.	Accession No.	Protein Description	Score	Mass [Da]	Matches	Coverage [%]	
1	gi|15010550	heat shock protein gp96 precursor	1179	90309	60	31,1	
2	gi|116256327	neprilysin	1176	86144	64	38,5	
6	gi|577295	KIAA0088	746	107158	34	24,6	glucosidase
8	gi|178536	aminopeptidase N precursor (EC 3.4.11.2)	630	109842	24	18,1	
9	gi|55743098	collagen alpha-3(VI) chain isoform 1 precursor	551	345167	20	6,4	
10	gi|68533085	MYO1C variant protein	550	125833	20	16,1	
11	gi|28334	unnamed protein product	540	103480	20	19,5	a-actinin
12	gi|306891	90kDa heat shock protein	532	83584	18	17,8	
14	gi|2804273	alpha actinin 4	456	102661	16	13,6	
15	gi|33636742	procollagen-lysine,2-oxoglutarate 5-dioxygenase 2 isoform 1 precursor	427	87784	14	14,2	
16	gi|11127638	GROS1-L protein	411	84216	13	15,8	
17	gi|119589620	hCG1646516	385	133590	8	7,7	S3-12, lipid droplet-binding
18	gi|19743813	integrin beta-1 isoform 1A precursor	373	91664	15	10,9	
19	gi|4503483	elongation factor 2	365	96246	11	10,8	
20	gi|915392	fatty acid synthase	356	275542	10	3,9	multi-enzyme, catalyzes the synthesis of palmitate from acetyl-CoA & malonyl-CoA to long-chain saturated fatty acids
21	gi|182855	80K-H protein	345	60228	19	13,9	Low Density Lipoprotein Receptor Class A domain cholesterol metabolism
22	gi|4186038	glucose 1-dehydrogenase	329	89393	10	10,6	
23	gi|32488	unnamed protein product	328	85020	8	10,9	histidine kinase-like ATPases
24	gi|4757756	annexin A2 isoform 2	318	38808	13	22,7	calcium-dependent phospholipid-binding protein
25	gi|10716563	calnexin precursor	317	67982	20	10,1	ER protein
26	gi|6424942	ALG-2 interacting protein 1	306	96646	9	9,8	mannosyltransferase
27	gi|37433	unnamed protein product	278	85304	10	10,7	transferrin receptor
28	gi|516764	motor protein	273	79830	9	12,9	
29	gi|28336	mutant beta-actin (beta'-actin)	259	42128	12	23,7	
30	gi|386758	GRP78 precursor, partial	242	72185	6	11,9	glucose-regulated protein
35	gi|307109	lysosomal membrane glycoprotein-1	201	45201	12	10,6	
36	gi|3299885	ES/130	197	109019	4	4,8	ribosome receptor
38	gi|3183975	MEMD protein	188	65614	7	11	cell adhesion molecule
39	gi|34339	LDL-receptor related precursor (AA -19 to 4525)	185	523119	7	1,4	central role in mammalian cholesterol metabolism; the receptor protein mammalian cholesterol metabolism
40	gi|13603394	type VI collagen alpha 2 chain precursor	184	109703	5	4	
46	gi|181184	stomatin peptide	128	31860	4	12,5	lipid raft-associated integral membrane protein
51	gi|6005942	transitional endoplasmic reticulum ATPase	114	89950	4	6,2	ER
62	gi|4758012	clathrin heavy chain 1	92	193260	2	1,3	involved in endocytosis
67	gi|6381989	adipocyte-derived leucine aminopeptidase	80	107689	3	2,4	ER peptidase
69	gi|15559717	AP2A1 protein	76	109096	2	1	involved in the formation of clathrin-coated pits
75	gi|799177	100 kDa coactivator	65	100313	2	2,1	lipid droplet-binding

A6

Hit No.	Accession No.	Protein Description	Score	Mass [Da]	Matches	Coverage [%]	
1	gi|16507237	78 kDa glucose-regulated protein precursor	1991	72402	120	50,6	
7	gi|862457	enoyl-CoA hydratase/3-hydroxyacyl-CoA dehydrogenase alpha-subunit of trifunctional protein	954	83648	43	31,8	fatty acid oxidation complex, mitochondrial
8	gi|4506675	dolichyl-diphosphooligosaccharide--protein glycosyltransferase subunit 1 precursor	864	68641	33	34,6	sugar transfer from dolichol-phosphate-sugars occurs exclusively on the luminal side of the ER
9	gi|292059	MTHSP75	819	74019	19	26,4	75kD glucose-regulated protein, mitochondrial hsp70
10	gi|5729877	heat shock cognate 71 kDa protein isoform 1	797	71082	28	28,3	
11	gi|20072835	ACSL1 protein, partial	588	60800	23	24,3	
12	gi|2851393	NADPH--cytochrome P450 reductase; Short=CPR; Short=P450R	585	77097	17	26,1	anchored to the ER membrane by its N-terminal hydrophobic region.
13	gi|4758304	protein disulfide-isomerase A4 precursor	540	73229	18	18,4	
14	gi|35218	unnamed protein product	532	76154	14	24,4	membrane-associated Ca2+-binding
15	gi|7212807	EH domain containing 2	521	61182	21	27,1	endocytosis, vesicle transport, and signal transduction
16	gi|21410323	Perilipin	507	56216	15	26,2	lipid droplet-binding
17	gi|4557735	amine oxidase [flavin-containing] A isoform 1	487	60157	16	28,3	mitochondrial enzyme

18	gi|4504505	peroxisomal multifunctional enzyme type 2 isoform 2	467	80092	11	19,7	involved in the peroxisomal beta-oxidation pathway for fatty acids
20	gi|5031631	lysosome membrane protein 2 isoform 1 precursor	438	54712	18	20,1	
21	gi|190074	lysyl hydroxylase	431	84098	11	16,6	
22	gi|12231182	65kDa FK506-binding protein	426	64777	13	13,1	derived from cells after 2-weeks retinoic acid (RA) induction
23	gi|3273228	very-long-chain acyl-CoA dehydrogenase	422	70848	14	17,1	catalyzes initial step of mitochondrial beta-oxidation of long-chain fatty acids with chain length of 14 to 20 carbons
24	gi|35493916	dolichyl-diphosphooligosaccharide--protein glycosyltransferase subunit 2 isoform 1 precursor	417	69355	13	25,8	sugar transfer from dolichol-phosphate-sugars occurs exclusively on the luminal side of the ER
25	gi|178536	aminopeptidase N precursor (EC 3.4.11.2)	289	109842	10	8,3	
26	gi|34758	unnamed protein product	279	85397	6	8,1	
27	gi|119625804	moesin, isoform CRA_b	274	66678	9	12,5	
28	gi|52545928	hypothetical protein	266	133214	7	8,1	alpha 3 type VI collagen isoform 5 precursor
29	gi|22761234	unnamed protein product	246	67434	11	10,3	
30	gi|4757756	annexin A2 isoform 2	245	38808	8	17,1	calcium-dependent phospholipid-binding protein
31	gi|3916257	FK506-binding protein	243	57583	11	13,7	superfamily of calcium sensors and calcium signal modulator
33	gi|4505467	5'-nucleotidase isoform 1 preproprotein	228	63898	8	12,9	plasma membrane protein
35	gi|28336	mutant beta-actin (beta'-actin)	223	42128	10	20,3	
36	gi|183008	glucocerebrosidase precursor (5' end put.); putative	207	57798	7	9,3	
37	gi|10716563	calnexin precursor	189	67982	5	9	ER
40	gi|4507677	endoplasmin precursor	173	92696	6	5,5	ER
46	gi|7657069	ERO1-like protein alpha precursor	126	55213	4	6,6	favors disulfide bond formation in the ER
47	gi|181184	stomatin peptide	122	31860	3	12,5	lipid raft-associated integral membrane protein
49	gi|984145	lanosterol synthase	113	67624	2	4,9	catalyzes cyclization of (S)-2,3 oxidosqualene to lanosterol, reaction forms sterol nucleus, ER membrane; peripheral
53	gi|35360	PDC-E2 precursor (AA -54 to 561)	102	65806	4	5,2	dihydrolipoamide acetyltransferase, mitochondrial
56	gi|15559516	Acyl-CoA synthetase family member 2	95	69035	2	4,2	needed for fatty acid synthesis, in mitochondrial matrix
69	gi|37848	unnamed protein product	69	14847	4	14,1	vimentin, head sequence - associated with perilipin
70	gi|809185	Chain A, Metal Binding On The Structure Of Annexin V And Implications For Membrane Binding	68	35840	2	8,5	Calcium PHOSPHOLIPID-Binding Protein
71	gi|15620821	KIAA1881 protein	68	133891	2	1,8	S3-12, lipid droplet-binding
77	gi|1373146	CALM	57	70879	2	4,9	clathrin assembly protein
85	gi|34339	LDL-receptor related precursor (AA -19 to 4525)	51	523119	4	0,4	central role in mammalian cholesterol metabolism; the receptor protein mammalian cholesterol metabolism

A7

Hit No.	Accession No.	Protein Description	Score	Mass [Da]	Matches	Coverage [%]	
1	gi|62414289	vimentin	1608	53676	112	65,5	intermediate filament - associated with perilipin
2	gi|31542947	60 kDa heat shock protein, mitochondrial	1495	61187	74	50,3	
3	gi|220702506	Chain A, TapasinERP57 HETERODIMER	1385	54541	85	55,5	protein disulfide isomerase, ER
4	gi|20070125	protein disulfide-isomerase precursor	1309	57480	91	61,2	
5	gi|19263767	Similar to cytoskeleton-associated protein 4, partial	1174	62194	42	53	
6	gi|4757810	ATP synthase subunit alpha, mitochondrial isoform a precursor	1120	59828	39	44,5	
11	gi|5174735	tubulin beta-4B chain	774	50255	43	38,7	
12	gi|4507729	tubulin beta-2A chain	681	50274	40	33,3	
13	gi|35505	pyruvate kinase	663	58411	24	36,2	
15	gi|21361322	tubulin beta-4A chain	618	50010	31	33,1	
18	gi|4240305	KIAA0908 protein	565	57134	24	29,1	
20	gi|1297274	beta-tubulin	548	50941	31	22	
21	gi|5453603	T-complex protein 1 subunit beta isoform 1	437	57794	11	24,1	Chaperon, protein folding in an ATP-dependent manner
22	gi|4557014	catalase	413	59947	14	26	
23	gi|703093	serine hydroxymethyltransferase, partial	398	52828	10	17,3	
24	gi|194388786	unnamed protein product	373	41544	13	23,1	highly similar to Polymerase I and transcript release factor
25	gi|159162689	Chain A, Human Protein Disulfide Isomerase, Nmr, 40 Structures	361	13363	21	58,3	
27	gi|23308577	D-3-phosphoglycerate dehydrogenase	320	57356	9	12,8	
28	gi|13276641	hypothetical protein	299	61919	7	12,7	M28 Zn-Peptidase Nicalin
29	gi|4757900	calreticulin precursor	298	48283	20	24,2	ER protein
30	gi|178390	aldehyde dehydrogenase	292	56858	11	17,6	
31	gi|4507813	UDP-glucose 6-dehydrogenase isoform 1	276	55674	9	15,2	
32	gi|45827806	atlastin-3	273	60960	13	20,5	dynamin-like GTPases involved in the generation of the tubular ER network
33	gi|10092665	sushi domain-containing protein 2 precursor	270	91746	9	10,3	contains glycosyl-phosphatidyl-inositol-anchored membrane family domains
34	gi|1263008	aldehyde dehydrogenase	267	57637	8	10,8	
36	gi|1136741	KIAA0002	250	59035	7	10,8	Chaperon, involved in protein folding
39	gi|433938	ERGIC53	241	57812	10	13,9	membrane protein of the ER-Golgi intermediate compartment
40	gi|1184537	thioredoxin reductase	236	55126	9	14,1	
41	gi|14017877	KIAA1830 protein	235	55511	6	11,5	TMX3, a thiol-disulfide oxidoreductase - disulfide bond formation in the endoplasmic reticulum
42	gi|793763	membrane-type matrix metalloproteinase	212	66183	7	10,1	
43	gi|4758868	prolyl 4-hydroxylase subunit alpha-2 isoform 1 precursor	196	61263	7	13,6	
44	gi|28336	mutant beta-actin (beta'-actin)	189	42128	7	20	
50	gi|4557303	fatty aldehyde dehydrogenase isoform 2	136	55269	5	8	converts long-chain fatty aldehydes to the corresponding acids
58	gi|3041771	perilipin	121	56201	3	7,3	lipid droplet-binding
59	gi|4507677	endoplasmin precursor	117	92696	3	4,9	
68	gi|4757756	annexin A2 isoform 2	98	38808	4	9,7	calcium-dependent phospholipid-binding protein
75	gi|181184	stomatin peptide	86	31860	2	9,7	lipid raft-associated integral membrane protein
81	gi|1575347	HU-K4	70	49196	2	4,3	phospholipase-D-like protein
91	gi|181575	dihydrolipoamide dehydrogenase precursor	54	54812	1	2,8	mitochondrial

94	gi|13375618	delta(24)-sterol reductase precursor	53	60803	3	3,1	participates in the biosynthesis of steroids
99	gi|5454084	serine palmitoyltransferase 1 isoform a	44	53281	1	3	involved in biosynthesis of sphingosine, precursor of many sphingolipids, anchored to the ER

A8

Hit No.	Accession No.	Protein Description	Score	Mass [Da]	Matches	Coverage [%]	
1	gi|32189394	ATP synthase subunit beta, mitochondrial precursor	1285	56525	86	63,1	
2	gi|28940	unnamed protein product	1283	57976	87	62	
3	gi|28940	serpin H1 precursor	1278	46525	85	57,7	localizes to the endoplasmic reticulum, molecular chaperone
4	gi|4757810	ATP synthase subunit alpha, mitochondrial isoform a precursor	1266	59828	54	48,8	
5	gi|1710248	protein disulfide isomerase-related protein 5	929	46512	39	44,4	
6	gi|340219	vimentin	831	53738	32	41,6	intermediate filament - associated with perilipin
7	gi|537526	microsomal epoxide hydrolase	799	53136	44	37,6	
8	gi|4503571	alpha-enolase isoform 1	758	47481	27	52,5	conversion of 2-phosphoglycerate (2-PG) to phosphoenolpyruvate (PEP), the ninth and penultimate step of glycolysis
9	gi|4929557	CGI-44 protein	626	50202	18	31,1	
10	gi|4504327	trifunctional enzyme subunit beta, mitochondrial precursor	598	51547	21	25,1	thioredoxin reductase
12	gi|473947	KIAA0115	517	50851	17	25,4	
15	gi|860986	protein disulfide isomerase	513	57043	22	36,6	
18	gi|31092	unnamed protein product	480	50437	29	24,2	elongation factor
19	gi|9836652	BSCv	461	47887	22	31,2	adipocyte plasma membrane-associated protein
20	gi|5821140	ASY	404	40422	21	24,4	
21	gi|4501885	actin, cytoplasmic 1	380	42052	15	32	
22	gi|4885281	glutamate dehydrogenase 1, mitochondrial precursor	380	61701	14	19,7	
23	gi|20067392	thioredoxin related protein	358	41361	22	26,7	
25	gi|35655	unnamed protein product	347	57458	14	23,8	
26	gi|6470150	BiP protein, partial	342	71002	9	16,7	autoantigen associated with rheumatoid arthritis
27	gi|18379349	synaptic vesicle membrane protein VAT-1 homolog	340	42122	15	24,7	abundant integral membrane protein, role of phospholipase D in the transport of VAT1 to plasma membranes
29	gi|499719	mitochondrial dihydrolipoamide succinyltransferase	307	49000	10	14,1	
30	gi|4503481	elongation factor 1-gamma	290	50429	11	16,9	
31	gi|24233517	inhibitor of nuclear factor kappa-B kinase-interacting protein isoform 1	289	43228	10	16,2	
32	gi|37492	alpha-tubulin	282	50810	10	22,2	
33	gi|40068518	6-phosphogluconate dehydrogenase, decarboxylating	256	53619	8	14,3	
35	gi|5453601	cartilage-associated protein precursor	246	47159	11	13,2	
38	gi|3043670	KIAA0573 protein	177	52282	7	7,5	ER protein, thioredoxin domains
39	gi|515634	ubiquinol-cytochrome c reductase core I protein	175	53270	5	10,8	
40	gi|4503529	eukaryotic initiation factor 4A-I isoform 1	169	46353	8	14,8	
41	gi|19743875	fumarate hydratase, mitochondrial	165	54773	5	10,8	
42	gi|409027	CDC42 GTPase-activating protein, partial	148	52966	5	10,4	Sec14p-like lipid-binding domain, phospholipid binding pocket
43	gi|4502679	CD63 antigen isoform A	145	26474	8	7,6	tetraspan family
44	gi|4507677	endoplasmin precursor	140	92696	3	4,2	ER protein
45	gi|4757900	calreticulin precursor	121	48283	5	17,3	ER protein
46	gi|4506675	dolichyl-diphosphooligosaccharide--protein glycosyltransferase subunit 1 precursor	111	68641	4	5,8	sugar transfer from dolichol-phosphate-sugars occurs exclusively on the luminal side of the ER
47	gi|4757756	annexin A2 isoform 2	111	38808	3	12,7	calcium-dependent phospholipid-binding protein
48	gi|181184	stomatin peptide	109	31860	4	10,8	lipid raft-associated integral membrane protein
50	gi|40254986	hydroxysteroid dehydrogenase-like protein 2 isoform 1	101	45651	4	6,7	
51	gi|3041771	perilipin	95	56201	3	4,6	lipid droplet-binding
55	gi|4557303	fatty aldehyde dehydrogenase isoform 2	86	55269	2	5,2	converts long-chain fatty aldehydes to the corresponding acids
67	gi|3095186	cargo selection protein TIP47	64	47175	2	3,9	lipid droplet-binding

A9

Hit No.	Accession No.	Protein Description	Score	Mass [Da]	Matches	Coverage [%]	
1	gi|4501885	actin, cytoplasmic 1	1367	42052	160	61,1	
2	gi|4757756	annexin A2 isoform 2	1285	38808	81	61,7	calcium-dependent phospholipid-binding protein
3	gi|4501881	actin, alpha skeletal muscle	941	42366	105	44,3	
7	gi|340219	vimentin	711	53738	21	33,9	intermediate filament - associated with perilipin
8	gi|31645	glyceraldehyde-3-phosphate dehydrogenase	667	36202	59	39,7	
9	gi|2906146	malate dehydrogenase precursor	608	35965	24	40,8	
12	gi|4503327	NADH-cytochrome b5 reductase 3 isoform 1	562	34441	27	40,5	cytoplasmic side of ER, functions in desaturation and elongation of fatty acids & cholesterol biosynthesis
13	gi|704416	elongation factor Tu	525	49851	17	32,1	
14	gi|4504505	peroxisomal multifunctional enzyme type 2 isoform 2	502	80092	14	20,1	peroxisomal beta-oxidation pathway for fatty acids
15	gi|4502101	annexin A1	478	38918	15	29,8	calcium-dependent phospholipid-binding protein
16	gi|5031857	L-lactate dehydrogenase A chain isoform 1	448	36950	13	31,9	
17	gi|5453543	aldo-keto reductase family 1 member C1	440	37221	14	37,8	conversion of aldehydes and ketones to their corresponding alcohols
19	gi|4557032	L-lactate dehydrogenase B chain	399	36900	12	28,7	
20	gi|509676	mitochondrial 3-oxoacyl-CoA thiolase	383	42469	13	33,8	
21	gi|35655	unnamed protein product	381	57458	15	22,4	protein disulfide isomerase (PDIa) family, ER, proteins involved in oxidative protein folding
22	gi|50592988	cytochrome b-c1 complex subunit 2, mitochondrial precursor	365	48584	12	28,3	
23	gi|4506667	60S acidic ribosomal protein P0	351	34423	10	28,4	
25	gi|1698401	VatI	343	32651	11	24	vesicle amine transport protein
26	gi|860986	protein disulfide isomerase	330	57043	8	14,9	
28	gi|31092	unnamed protein product	312	50437	14	19,5	elongation factor

29	gi|386758	GRP78 precursor, partial	312	72185	7	14,5	glucose-regulated protein
30	gi|12053327	hypothetical protein	309	44860	12	33,5	transmembrane protein 43 (LUMA)
32	gi|6005721	erlin-2 isoform 1	278	38044	7	16,8	localized to lipid rafts of the endoplasmic reticulum
33	gi|3288815	citrate synthase	267	51959	9	14,8	
34	gi|5821140	ASY	256	40422	11	17,4	
35	gi|793763	membrane-type matrix metalloproteinase	254	66183	9	10,1	
36	gi|28626510	reticulocalbin-3 precursor	249	37470	9	15,5	calcium-binding protein
37	gi|62898103	aspartate aminotransferase 2 precursor variant	246	47916	5	14	
38	gi|4505763	phosphoglycerate kinase 1	242	44985	9	21,3	catalyzes the conversion of 1,3-diphosphoglycerate to 3-phosphoglycerate
39	gi|2337920	syntaxin 7	240	29941	4	21,5	proteins implicated in the docking of synaptic vesicles with the presynaptic plasma membrane
40	gi|4503143	cathepsin D preproprotein	233	45037	10	16,7	protease
42	gi|28178832	isocitrate dehydrogenase [NADP], mitochondrial precursor	225	51333	6	14,8	
44	gi|499158	mitochondrial acetoacetyl-CoA thiolase	224	45537	11	16,2	catalyzation of the reversible thiolytic cleavage of 3-ketoacyl-CoA into acyl-CoA and acetyl-CoA
50	gi|181184	stomatin peptide	210	31860	5	19,1	lipid raft-associated integral membrane protein
55	gi|10716563	calnexin precursor	177	67982	6	8,4	ER protein
57	gi|8393516	sterol-4-alpha-carboxylate 3-dehydrogenase, decarboxylating	173	42159	5	11,5	protein is localized in ER and is involved in cholesterol biosynthesis
68	gi|5031765	corticosteroid 11-beta-dehydrogenase isozyme 1	136	32608	4	13	catalyzes the conversion of the stress hormone cortisol to the inactive metabolite cortisone (and reverse) *
72	gi|1743867	acid ceramidase	131	45077	5	7,8	catalyzes the hydrolysis of ceramide into sphingosine and free fatty acid
89	gi|546518	stearoyl-CoA desaturase	96	27713	2	8	key enzyme in fatty acid metabolism, responsible for forming a double bond in stearoyl-CoA

* Too much cortisol can lead to central obesity.

A10

Hit No.	Accession No.	Protein Description	Score	Mass [Da]	Matches	Coverage [%]	
1	gi|4757756	annexin A2 isoform 2	1001	38808	61	55,5	calcium-dependent phospholipid-binding protein
2	gi|181184	stomatin peptide	910	31860	63	46,9	lipid raft-associated integral membrane protein
6	gi|4501885	actin, cytoplasmic 1	690	42052	45	42,9	
7	gi|4503327	NADH-cytochrome b5 reductase 3 isoform 1	690	34441	60	40,5	membrane-bound cytoplasmic ER side, functions in desaturation & elongation of fatty acids, in cholesterol biosynthesis
8	gi|119609105	prohibitin 2	688	32262	24	50,2	lipid raft-associated integral membrane protein
10	gi|238427	Porin 31HM [human, skeletal muscle membranes, Peptide, 282 aa]	626	30737	32	53,9	beta barrel proteins that cross a cellular membrane and act as a pore through which molecules can diffuse
11	gi|4505773	prohibitin	568	29843	21	42,3	lipid raft-associated integral membrane protein
12	gi|15928608	Solute carrier family 25 (mitochondrial carrier; adenine nucleotide translocator), member 6	505	33133	22	34,2	
13	gi|45829841	SLC25A5 protein, partial	491	35499	23	28,5	ADP/ATP transporter, mitochondrial carrier
14	gi|4503143	cathepsin D preproprotein	466	45037	27	23,3	protease, most of the family members become activated at the low pH found lysosomes
15	gi|2906146	malate dehydrogenase precursor	464	35965	19	29,9	
16	gi|7705855	estradiol 17-beta-dehydrogenase 12	448	34416	20	26,9	enzyme converts estrone into estradiol in ovarian tissue - also involved in fatty acid elongation
17	gi|190200	porin, partial	394	38639	15	28,8	beta barrel proteins that cross a cellular membrane and act as a pore through which molecules can diffuse
18	gi|38649415	Signal recognition particle receptor, B subunit	390	29926	10	29,9	protein targeting to the membrane of the ER
19	gi|5803013	endoplasmic reticulum resident protein 29 isoform 1 precursor	376	29032	16	28,4	ER protein
20	gi|5031857	L-lactate dehydrogenase A chain isoform 1	364	36950	12	25,9	
22	gi|28940	unnamed protein product	311	57976	9	17,4	ATP synthase, mitochondrial
24	gi|35903	ribosomal protein L7	287	29221	14	30,6	
26	gi|15680023	B-cell receptor-associated protein 31	280	28032	15	22	
27	gi|16877071	ATP synthase, H+ transporting, mitochondrial F1 complex, gamma polypeptide 1	279	33002	9	22,5	
29	gi|4885079	ATP synthase subunit gamma, mitochondrial isoform H (heart) precursor	259	32917	9	22,2	
31	gi|5803225	14-3-3 protein epsilon	251	29326	9	21,2	mediation of signal transduction by binding phosphoserine-containing proteins, activities e.g. regulation of insulin sensitivity
32	gi|189617	protein PP4-X	246	36262	7	13,4	member of the phospholipase A2 inhibitor group, annexin domains
33	gi|25188179	voltage-dependent anion-selective channel protein 3 isoform 1	244	30981	9	14,1	beta barrel proteins that cross a cellular membrane and act as a pore through which molecules can diffuse
34	gi|306785	G protein beta subunit	242	38061	13	17,6	pre-mRNA processing and cytoskeleton assembly
37	gi|4502013	adenylate kinase 2, mitochondrial isoform a	231	26689	8	27,6	
38	gi|950004	citrate transporter protein	225	34405	11	16,4	
39	gi|4507953	14-3-3 protein zeta/delta	221	27899	11	18	mediation of signal transduction by binding phosphoserine-containing proteins, activities e.g. regulation of insulin sensitivity
42	gi|31645	glyceraldehyde-3-phosphate dehydrogenase	220	36202	9	24,8	
44	gi|4240305	KIAA0908 protein	215	57134	9	13	prenylcysteine lyase, oxidoreductase, specifically acting on a sulfur group
45	gi|6470150	BiP protein, partial	209	71002	6	11,4	glucose-regulated protein (grp78), ER chaperonin
49	gi|9622124	androgen-regulated short-chain dehydrogenase/reductase 1	204	35791	4	17,6	
57	gi|1575000	2,4-dienoyl-CoA reductase	173	36096	7	15,8	enzyme which participates in the beta oxidation and metabolism of unsaturated fatty enoyl-CoA esters
58	gi|35655	unnamed protein product	170	57458	6	10,8	protein disulfide isomerase (PDIa), involved in oxidative protein folding in the ER
59	gi|178849	apolipoprotein E	168	36302	6	10,4	transports lipoproteins, fat-soluble vitamins, and cholesterol- normally in chylomicrons
66	gi|6330243	KIAA1181 protein	147	37312	5	10,7	endoplasmic reticulum-Golgi intermediate compartment (ERGIC)
67	gi|16924265	Enoyl Coenzyme A hydratase 1, peroxisomal	143	36078	2	9,5	enzyme hydrates the double bond between second and third carbons on acyl-CoA, essential to metabolizing FAs
68	gi|2078327	3-hydroxyacyl-CoA dehydrogenase, partial	143	33608	8	11,7	participates in fatty acid elongation and fatty acid metabolism
69	gi|4502101	annexin A1	142	38918	6	11	calcium-dependent phospholipid-binding protein
83	gi|15826034	Chain A, Apolipoprotein E3 22kd Fragment Lys146gln Mutant	118	22174	5	17,3	lipid binding protein
96	gi|2258418	dolichol monophosphate mannose synthase	96	28857	3	13	sugar transfer from dolichol-phosphate-sugars occurs exclusively on the luminal side of the ER
103	gi|5031765	corticosteroid 11-beta-dehydrogenase isozyme 1	84	32608	4	7,2	catalyzes the conversion of the stress hormone cortisol to the inactive metabolite cortisone (and reverse) *
116	gi|179832	calnexin	67	67948	1	2,7	ER protein
120	gi|546518	stearoyl-CoA desaturase	62	27713	2	8	key enzyme in fatty acid metabolism, responsible for forming a double bond in stearoyl-CoA

A11

Hit No.	Accession No.	Protein Description	Score	Mass [Da]	Matches	Coverage [%]

3	gi|34147513	ras-related protein Rab-7a	700	23760	35	69,1	
6	gi|350476	protein NIG58,Bence-Jones	566	23061	76	36,7	
7	gi|13569962	ras-related protein Rab-1B	548	22328	40	61,7	
8	gi|351205	protein NIG64 lambda,Bence-Jones	546	22946	66	36,6	
9	gi|4501885	actin, cytoplasmic 1	532	42052	29	40	
11	gi|508285	Rab5c-like protein	523	23781	22	51,4	
14	gi|550062	GTP-binding protein	459	23720	14	44,3	
15	gi|4504517	heat shock protein beta-1	455	22826	20	43,4	
19	gi|148596959	redox-regulatory protein FAM213A isoform 1 precursor	380	25861	19	31,4	
20	gi|4505591	peroxiredoxin-1	368	22324	15	31,2	
21	gi|4502303	ATP synthase subunit O, mitochondrial precursor	358	23377	15	35,2	
22	gi|19923231	ras-related protein Rab-6A isoform a	356	23648	15	39,4	
23	gi|19923262	ras-related protein Rab-5A	343	23872	11	34	
24	gi|20147741	Ras family small GTP binding protein TC21	336	23508	11	34,5	
25	gi|15451856	caveolin-1 isoform alpha	335	20630	18	43,8	envolved in endocytosis
26	gi|37138	unnamed protein product	328	133321	8	6,6	
27	gi|5453549	peroxiredoxin-4 precursor	323	30749	15	28,8	
29	gi|181184	stomatin peptide	317	31860	6	20,1	lipid raft-associated integral membrane protein
30	gi|6647832	Membrane-associated progesterone receptor component 2; Steroid receptor protein DG6	315	23861	12	25,1	
31	gi|425518	anti-colorectal carcinoma heavy chain	314	51254	32	12	
32	gi|2852648	unknown	312	22435	15	39,7	
33	gi|10880989	ras-related protein Rab-18 isoform 1	302	23248	8	36,4	lipid droplet-binding
35	gi|4506371	ras-related protein Rab-5B isoform 1	295	23920	10	34	
36	gi|7643782	HDCMD47P	294	25683	12	25,2	
37	gi|136066	Triosephosphate isomerase; Short=TIM; Triose-phosphate isomerase	290	26894	9	29,4	
38	gi|793763	membrane-type matrix metalloproteinase	288	66183	18	12,9	
41	gi|763130	YPT3	274	24673	10	24,3	
42	gi|4757756	annexin A2 isoform 2	272	38808	8	23,9	calcium-dependent phospholipid-binding protein
43	gi|1922287	enoyl-CoA hydratase	269	31807	10	26,6	
44	gi|5454028	ras-related protein R-Ras precursor	267	23637	9	23,4	
45	gi|5729875	membrane-associated progesterone receptor component 1	260	21772	16	24,1	
46	gi|19923437	GTP:AMP phosphotransferase, mitochondrial isoform a	253	25550	10	30,4	
47	gi|7661678	ras-related protein Rap-1b isoform 1 precursor	245	21040	12	28,8	
48	gi|34709	manganese superoxide dismutase (MnSOD)	224	24891	9	20,3	
49	gi|5802974	thioredoxin-dependent peroxide reductase, mitochondrial isoform a precursor	224	28017	11	14,5	
52	gi|7706563	ras-related protein Rab-8B	212	23740	13	18,4	
53	gi|234746	RAS-related protein MEL	206	23753	13	18,4	
54	gi|4758504	3-hydroxyacyl-CoA dehydrogenase type-2 isoform 1	200	27134	7	28,4	
75	gi|4757900	calreticulin precursor	133	48283	5	9,8	ER
82	gi|340219	vimentin	114	53738	2	5,2	intermediate filament - associated with perilipin
112	gi|458119	acyl-CoA oxidase	62	74910	1	2	

A12

Hit No.	Accession No.	Protein Description	Score	Mass [Da]	Matches	Coverage [%]	
4	gi|181250	cyclophilin, partial	830	22654	60	69,6	
6	gi|28317	unnamed protein product	506	59720	23	21,6	
8	gi|177870	alpha-2-macroglobulin precursor	311	164600	30	2,5	
9	gi|2852648	unknown	296	22435	9	36,1	
10	gi|4757756	annexin A2 isoform 2	288	38808	12	18	calcium-dependent phospholipid-binding protein
11	gi|6647832	Membrane-associated progesterone receptor component 2; Steroid receptor protein DG6	284	23861	8	22,4	
12	gi|15451856	caveolin-1 isoform alpha	283	20630	13	47,8	envolved in endocytosis
14	gi|9945306	microsomal glutathione S-transferase 1 isoform a	262	17644	13	31	
15	gi|182516	ferritin light subunit, partial	258	16441	15	44,1	
16	gi|28375485	unnamed protein product	252	25790	13	24	
17	gi|28336	mutant beta-actin (beta'-actin)	247	42128	11	18,1	
19	gi|425518	anti-colorectal carcinoma heavy chain	224	51254	8	12	
20	gi|5454090	translocon-associated protein subunit delta isoform 2 precursor	222	19158	12	28,3	
21	gi|4506691	40S ribosomal protein S16	222	16549	7	32,2	
22	gi|4506685	40S ribosomal protein S13	216	17212	9	35,1	
23	gi|4506597	60S ribosomal protein L12	215	17979	6	33,3	
24	gi|4502205	ADP-ribosylation factor 4	215	20612	9	27,2	
25	gi|5031635	cofilin-1	214	18719	10	42,2	
27	gi|4758714	microsomal glutathione S-transferase 3	211	16734	7	18,4	
56	gi|181184	stomatin peptide	118	31860	4	11,1	lipid raft-associated integral membrane protein
65	gi|303618	phospholipase C-alpha	110	57065	3	6,3	
95	gi|179832	calnexin	69	67948	1	2,7	
107	gi|4505893	proteolipid protein 2	58	17022	2	8,6	
123	gi|5729875	membrane-associated progesterone receptor component 1	44	21772	1	7,2	

A13

Hit No.	Accession No.	Protein Description	Score	Mass [Da]	Matches	Coverage [%]	
4	gi|28317	unnamed protein product	345	59720	14	15,3	
6	gi|9945306	microsomal glutathione S-transferase 1 isoform a	223	17644	28	31	
10	gi|303618	phospholipase C-alpha	152	57065	7	12,5	
21	gi|4757756	annexin A2 isoform 2	93	38808	5	10	calcium-dependent phospholipid-binding protein
29	gi|181184	stomatin peptide	68	31860	2	6,9	lipid raft-associated integral membrane protein

Table S2b

IP : Poietics AIM **VIM 3B4**

B1

Hit No.	Accession No.	Protein Description	Score	Mass [Da]	Matches	Coverage [%]	
8	gi|4972627	caveolin 1	66	13237	2	18,6	involved in endocytosis

B2

Hit No.	Accession No.	Protein Description	Score	Mass [Da]	Matches	Coverage [%]	
8	gi|61743954	neuroblast differentiation-associated protein AHNAK isoform 1	837	629213	40	5,6	AHNAK binds annexin and works together in the developement of intracellar membranes
17	gi|15620821	KIAA1881 protein	121	133891	3	2,9	S3-12, lipid droplet-binding
18	gi|34339	LDL-receptor related precursor (AA -19 to 4525)	118	523119	5	0,9	plays a central role in mammalian cholesterol metabolism
27	gi|915392	fatty acid synthase	83	275542	3	1,3	multi enzyme, catalyses synthesis of palmitate from acetyl-CoA & malonyl-CoA to long-chain saturated fatty acid
30	gi|181184	stomatin peptide	73	31860	2	5,2	lipid raft-associated integral membrane protein
32	gi|4757756	annexin A2 isoform 2	71	38808	2	6,8	calcium-dependent phospholipid-binding protein

B3

Hit No.	Accession No.	Protein Description	Score	Mass [Da]	Matches	Coverage [%]	
2	gi|61743954	neuroblast differentiation-associated protein AHNAK isoform 1	688	629213	27	4,8	AHNAK binds annexin and works together in the developement of intracellar membranes
5	gi|68533031	FASN variant protein	608	279819	16	5,5	polymerize simple fatty acids into a large variety of different products, fatty acid synthase
7	gi|7305053	myoferlin isoform a	532	236100	19	7,6	calcium/phospholipid-binding protein
11	gi|15620821	KIAA1881 protein	300	133891	7	6,2	S3-12, lipid droplet-binding
16	gi|34339	LDL-receptor related precursor (AA -19 to 4525)	225	523119	7	1,6	plays a central role in mammalian cholesterol metabolism
19	gi|35655	unnamed protein product	152	57458	6	11	disuldide isomerase, involved in oxidative protein folding in the ER
24	gi|4757756	annexin A2 isoform 2	110	38808	4	6,8	calcium-dependent phospholipid-binding protein
30	gi|181184	stomatin peptide	81	31860	2	6,9	lipid raft-associated integral membrane protein
34	gi|4758012	clathrin heavy chain 1	58	193260	2	1,4	involved in endocytosis

B4

Hit No.	Accession No.	Protein Description	Score	Mass [Da]	Matches	Coverage [%]	
12	gi|68533031	FASN variant protein	272	279819	7	2,6	polymerize simple fatty acids into a large variety of different products, fatty acid synthase
16	gi|4757756	annexin A2 isoform 2	245	38808	8	18,6	calcium-dependent phospholipid-binding protein
21	gi|5453832	hypoxia up-regulated protein 1 precursor	185	111494	10	7,8	endoplasmic reticulum (ER) under hypoxic conditions
33	gi|34339	LDL-receptor related precursor (AA -19 to 4525)	109	523119	5	1	plays a central role in mammalian cholesterol metabolism
45	gi|799177	100 kDa coactivator	57	100313	2	2,1	lipid droplet-binding protein

B5

Hit No.	Accession No.	Protein Description	Score	Mass [Da]	Matches	Coverage [%]	
6	gi|6470150	BiP protein, partial	576	71002	16	25,2	glucose-regulated protein (grp78), ER chaperone
9	gi|577295	KIAA0088	407	107158	18	15,3	ER protein, glycosidase
13	gi|10716563	calnexin precursor	297	67982	12	10,1	ER protein
15	gi|182855	80K-H protein	277	60228	14	10,4	contains low density lipoprotein receptor class A domain, cholesterol metabolism
27	gi|1049053	encodes region of fatty acid synthase activity; FAS; multifunctional protein	172	275553	5	1,8	
32	gi|4757756	annexin A2 isoform 2	135	38808	4	8,8	calcium-dependent phospholipid-binding protein
37	gi|6424942	ALG-2 interacting protein 1	124	96646	3	4,1	mannosyltransferase in ER
45	gi|6005942	transitional endoplasmic reticulum ATPase	84	89950	3	5,2	ER protein
49	gi|34339	LDL-receptor related precursor (AA -19 to 4525)	75	523119	3	0,6	plays a central role in mammalian cholesterol metabolism
51	gi|15559717	AP2A1 protein	72	109096	1	1	involved in the formation of clathrin-coated pits

B6

Hit No.	Accession No.	Protein Description	Score	Mass [Da]	Matches	Coverage [%]	
4	gi|62414289	vimentin	871	53676	35	45,3	intermediate filament - associated with perilipin
8	gi|862457	enoyl-CoA hydratase/3-hydroxyacyl-CoA dehydrogenase α-subunit of trifunctional protein	579	83648	20	22,7	hydrates the double bond between the second & third carb ons onacyl- CoA, essential to metabolizing fatty acids
10	gi|4506675	dolichyl-diphosphooligosaccharide--protein glycosyltransferase subunit 1 precursor	490	68641	19	21,4	sugar transfer from dolichol-phosphate -sugars occurs exclusively on the luminal side of ER
11	gi|20072835	ACSL1 protein, partial	474	60800	19	19,7	adipocytokine pathway
13	gi|179930	carboxylesterase, partial	413	56446	8	18,1	
16	gi|2851393	RecName: Full=NADPH--cytochrome P450 reductase; Short=CPR; Short=P450R	352	77097	10	19,4	cytoplasmic side of ER, functions in desaturation and elongation of fatty acids
18	gi|35218	unnamed protein product	348	76154	11	19,2	membrane-associated Ca2+-binding protein p68, annexin domains
22	gi|183008	glucocerebrosidase precursor (5' end put.); putative	298	57798	9	12	
38	gi|3273228	very-long-chain acyl-CoA dehydrogenase	154	70848	4	8,2	catalizes the initial step of mitochondrial beta-oxidation of long-chain fatty acids
47	gi|303618	phospholipase C-alpha	108	57065	3	6,1	
66	gi|3041771	perilipin	59	56201	2	2,7	lipid-droplet-binding

B7

Hit No.	Accession No.	Protein Description	Score	Mass [Da]	Matches	Coverage [%]	
1	gi|62414289	vimentin	1676	53676	93	62	intermediate filament - associated with perilipin
3	gi|220702506	Chain A, TapasinERP57 HETERODIMER	897	54541	36	44,7	catalyzing the formation of disulfide bonds of newly synthesized polypeptides in the ER
4	gi|35655	unnamed protein product	845	57458	40	42,7	protein disulfide isomerase (PDIa), involved in oxidative protein folding in the ER
11	gi|1710248	protein disulfide isomerase-related protein 5	412	46512	16	18,8	ER protein
21	gi|159162689	Chain A, Human Protein Disulfide Isomerase, Nmr, 40 Structures	256	13363	7	42,5	ER protein
22	gi|4757900	calreticulin precursor	194	48283	7	12	ER protein
26	gi|194388786	unnamed protein product	143	41544	5	10,5	prenylcysteine lyase

B8

Hit No.	Accession No.	Protein Description	Score	Mass [Da]	Matches	Coverage [%]	
1	gi|62414289	vimentin	1532	53676	87	63,5	intermediate filament - associated with perilipin
3	gi|32454741	serpin H1 precursor	1127	46525	72	61,5	localizes to the endoplasmic reticulum, molecular chaperone
8	gi|18379349	synaptic vesicle membrane protein VAT-1 homolog	444	42122	20	26,2	abundant integral membrane protein, role of phospholipase D in the transport of VAT1 to plasma membranes
13	gi|9836652	BSCv	326	47887	11	19,8	adipocyte plasma membrane-associated protein
19	gi|1710248	protein disulfide isomerase-related protein 5	273	46512	6	15,9	
26	gi|860986	protein disulfide isomerase	197	57043	6	15,8	
35	gi|3043670	KIAA0573 protein	157	52282	5	5,5	ER protein
53	gi|3095186	cargo selection protein TIP47	76	47175	2	3,9	lipid droplet-binding
62	gi|5031699	flotillin-1	56	47554	2	4,2	
63	gi|181184	stomatin peptide	56	31860	1	2,8	lipid raft-associated integral membrane protein
64	gi|4757756	annexin A2 isoform 2	55	38808	3	9,7	calcium-dependent phospholipid-binding protein

B9

Hit No.	Accession No.	Protein Description	Score	Mass [Da]	Matches	Coverage [%]	
1	gi|4757756	annexin A2 isoform 2	1357	38808	85	62,8	calcium-dependent phospholipid-binding protein
6	gi|4503327	NADH-cytochrome b5 reductase 3 isoform 1	522	34441	32	33,2	cytoplasmic side of the ER, functions in desaturation and elongation of fatty acids
12	gi|119609105	prohibitin 2	420	32262	12	41,2	lipid raft-associated integral membrane protein
13	gi|340219	vimentin	420	53738	16	29,2	intermediate filament - associated with perilipin
15	gi|4502101	annexin A1	351	38918	11	26,9	calcium-dependent phospholipid-binding protein
48	gi|6005721	erlin-2 isoform 1	131	38044	3	7,1	localized to lipid rafts of the endoplasmic reticulum
51	gi|8393516	sterol-4-alpha-carboxylate 3-dehydrogenase, decarboxylating	115	42159	3	11,8	
53	gi|178849	apolipoprotein E	109	36302	4	10,4	transports lipoproteins, fat-soluble vitamins and cholesterol - normally in chylomicrons
56	gi|5031765	corticosteroid 11-beta-dehydrogenase isozyme 1	100	32608	3	7,2	
57	gi|1743867	acid ceramidase	100	45077	3	5,8	
58	gi|15826034	Chain A, Apolipoprotein E3 22kd Fragment Lys146gln Mutant	98	22174	4	17,3	transports lipoproteins, fat-soluble vitamins and cholesterol - normally in chylomicrons
59	gi|181184	stomatin peptide	97	31860	2	6,9	lipid raft-associated integral membrane protein
92	gi|179832	calnexin	41	67948	1	2,7	ER protein

B10

Hit No.	Accession No.	Protein Description	Score	Mass [Da]	Matches	Coverage [%]	
7	gi|181184	stomatin peptide	719	31860	41	41,7	lipid raft-associated integral membrane protein
18	gi|809185	Chain A, Metal Binding On The Structure Of Annexin V And Implications For Membrane Binding	500	35840	16	30,1	
25	gi|6647832	Membrane-associated progesterone receptor component 2; Steroid receptor protein DG6	279	23861	5	22,4	steroid receptor protein
28	gi|4505773	prohibitin	247	29843	6	23,5	lipid raft-associated integral membrane protein
32	gi|1020320	CDM protein	152	14066	7	18,9	ER-mitochondrial interface
48	gi|5803013	endoplasmic reticulum resident protein 29 isoform 1 precursor	101	29032	3	6,5	ER protein
50	gi|4757756	annexin A2 isoform 2	85	38808	4	6,2	calcium-dependent phospholipid-binding protein
58	gi|340219	vimentin	62	53738	1	2,1	intermediate filament - associated with perilipin
60	gi|181557	NADH cytochrome b5 reductase (EC 1.6.2.2), partial	60	31394	1	4	cytoplasmic side of the ER, functions in desaturation and elongation of fatty acids

B11

Hit No.	Accession No.	Protein Description	Score	Mass [Da]	Matches	Coverage [%]	
20	gi|2852648	unknown	485	22435	19	47,4	chaperon, suppresses protein aggregation, protein processing in ER
40	gi|15451856	caveolin-1 isoform alpha	190	20630	8	25,8	involved in endocytosis
42	gi|340219	vimentin	183	53738	6	12,2	intermediate filament - associated with perilipin
47	gi|6857802	ER lumen protein retaining receptor 3 isoform a	136	25238	4	18,7	ER protein
52	gi|5729875	membrane-associated progesterone receptor component 1	111	21772	5	15,9	steroid receptor protein
63	gi|181557	NADH cytochrome b5 reductase (EC 1.6.2.2), partial	77	31394	1	4	cytoplasmic side of the ER, functions in desaturation and elongation of fatty acids
66	gi|6647832	Membrane-associated progesterone receptor component 2; Steroid receptor protein DG6	70	23861	2	12,6	steroid receptor protein
68	gi|181184	stomatin peptide	67	31860	1	2,8	lipid raft-associated integral membrane protein
82	gi|10880989	ras-related protein Rab-18 isoform 1	47	23248	1	5,3	lipid droplet-binding

B12

Hit No.	Accession No.	Protein Description	Score	Mass [Da]	Matches	Coverage [%]	
28	gi|340219	vimentin	173	53738	8	13,3	intermediate filament - associated with perilipin
35	gi|4757756	annexin A2 isoform 2	155	38808	6	10	calcium-dependent phospholipid-binding protein
41	gi|4972627	caveolin 1	126	13237	4	37,2	
75	gi|181184	stomatin peptide	59	31860	2	2,8	lipid raft-associated integral membrane protein
82	gi|4505893	proteolipid protein 2	52	17022	2	8,6	

B13

Hit No.	Accession No.	Protein Description	Score	Mass [Da]	Matches	Coverage [%]	
39	gi|4757756	annexin A2 isoform 2	47	38808	2	6,8	calcium-dependent phospholipid-binding protein
